# Application of magnetism in tissue regeneration: recent progress and future prospects

**DOI:** 10.1093/rb/rbae048

**Published:** 2024-05-07

**Authors:** Wenchao Guan, Hongxia Gao, Yaqiong Liu, Shaolan Sun, Guicai Li

**Affiliations:** Key Laboratory of Neuroregeneration, Co-innovation Center of Neuroregeneration, Nantong University, Nantong 226001, China; Key Laboratory of Neuroregeneration, Co-innovation Center of Neuroregeneration, Nantong University, Nantong 226001, China; Key Laboratory of Neuroregeneration, Co-innovation Center of Neuroregeneration, Nantong University, Nantong 226001, China; Key Laboratory of Neuroregeneration, Co-innovation Center of Neuroregeneration, Nantong University, Nantong 226001, China; Key Laboratory of Neuroregeneration, Co-innovation Center of Neuroregeneration, Nantong University, Nantong 226001, China; State Key Laboratory of Polymer Materials Engineering, Sichuan University, Chengdu 610065, China

**Keywords:** tissue regeneration, magnetic fields, magnetic materials, cell behavior, magnetic regulation

## Abstract

Tissue regeneration is a hot topic in the field of biomedical research in this century. Material composition, surface topology, light, ultrasonic, electric field and magnetic fields (MFs) all have important effects on the regeneration process. Among them, MFs can provide nearly non-invasive signal transmission within biological tissues, and magnetic materials can convert MFs into a series of signals related to biological processes, such as mechanical force, magnetic heat, drug release, etc. By adjusting the MFs and magnetic materials, desired cellular or molecular-level responses can be achieved to promote better tissue regeneration. This review summarizes the definition, classification and latest progress of MFs and magnetic materials in tissue engineering. It also explores the differences and potential applications of MFs in different tissue cells, aiming to connect the applications of magnetism in various subfields of tissue engineering and provide new insights for the use of magnetism in tissue regeneration.

## Graphical Abstract

**Figure:**
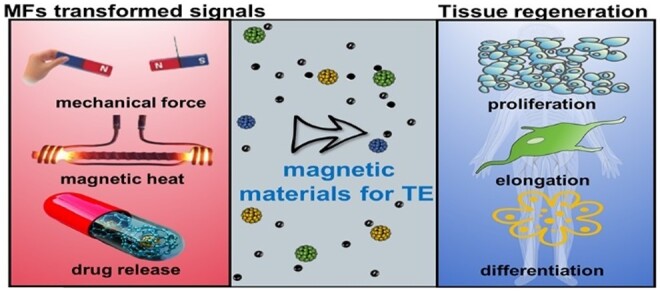

## Introduction

Tissue engineering is one of the research hotspots in the biomedical field in the 21st century [1–3]. Generally, tissue regeneration aims to enhance the body’s repair and regeneration processes by investigating the mechanisms of growth and development, as well as the structural characteristics of tissues, both in normal physiological states and after injury [4]. The ultimate objective is to regenerate tissues and organs that are indistinguishable from their pre-injury state [5]. A certain lower eukaryotes, including salamanders [6], leeches and earthworms, possess remarkable regenerative abilities. Even after sustaining injuries, these organisms can regenerate damaged internal organs and grow new limbs that exhibit both proper form and function. However, high-grade animals, particularly humans, have only limited regenerative capacities, primarily confined to the liver, blood and epidermis [7]. When humans are seriously damaged by tissues and organs, they will at least cause mobility difficulties, affect the quality of life and in severe cases, they may completely lose their ability for work, bringing heavy burdens to families and society and even losing their lives [8, 9].

Currently, there are three primary strategies for treating tissue and organ damage. The first approach involves allogeneic or even xenotransplantation, where organs or tissues are transplanted from donors [10, 11]. However, this approach raises some issues that require urgent attention, such as the source of donations, ethics, immune exclusion, etc. The second strategy involves the use of artificial organs. Although these artificial organs can partially perform certain functions of the original organs, they are still far from being able to fully replace autologous organs. The third strategy involves repairing damaged tissues and organs through tissue engineering materials or drugs (bioactive molecules that have a positive effect on tissue regeneration, such as anti-inflammatory, antibacterial, and promoting cell growth) [12, 13]. While this approach may not yet be capable of fully regenerating a perfect organ, significant progress has been achieved in this field [14–16].

Extensive studies have revealed that the type, morphology and surface characteristics of materials play a significant role in tissue regeneration [17–21]. Furthermore, external factors such as light, ultrasound, electric fields and MFs display diverse effects on the repair of damaged tissues [22–29]. Cells possess the ability to sense various chemical, electrical, thermal and mechanical signals [30–33]. Given that biological substances exhibit negligible magnetic permeability, deep stimulation *in vivo* through MFs has gained attention [34]. The manipulation of cells using MFs has emerged as a promising technique in life science these years [35, 36]. This approach relies on carriers that convert MFs into signals which can be sensed by bioreceptors in cells, including intracellular magnetically induced proteins and various magnetic materials. MFs and magnetic materials can exert different effects on cells, such as magnetocaloric, magnetochemical and magnetostrictive effect [37].

In this review, the definition, classification and latest progress of MFs and magnetic materials in tissue regeneration from the aspects of MFs and magnetic materials were mainly introduced, the differences and possible application principles in different tissue cells of MFs and magnetic materials were discussed. Further on, the biophysical challenges and future opportunities were also put forward. The present study is aim to summarize the development of magnetism in various subdivisions of tissue engineering and provide new perspectives for the application of magnetism in tissue regeneration.

## Introduction to MFs

All living things on Earth are exposed to the Earth’s magnetic field (MF) [38], which not only serves as a source of information about the direction of some organisms but also produces unique biological effects at the cellular level [39, 40]. MFs’ strength and direction are typically determined by a Gaussian meter, and measurements and visualization of three-dimensional radial and vector MFs distributions based on magnetic computed tomography (CT) methods have been reported [41]. Applying this method, a simple probe can be used to measure the surrounding 3D MFs, such as a spherical area. Studies have shown that using spherical CT probes and CT reconstruction algorithms, the MFs distribution of the object can be seen intuitively from the measurement and calculation results [41]. In addition to using various methods to directly measure calculations, a variety of software such as COMSOL, ANSYS, etc. have been developed and models have been established to simulate the MFs distribution [42]. For example, Rakotoarison *et al.* [43] used the Coulometric method to describe a new expression for scalar potential and generated MFs by radially polarized magnets, which could be used to calculate the MFs at the area around the magnet. Advances in detection and simulation technology will further advance the design of magnetic stimulation systems, which is expected to improve the accuracy of MFs.

**Figure 1.**
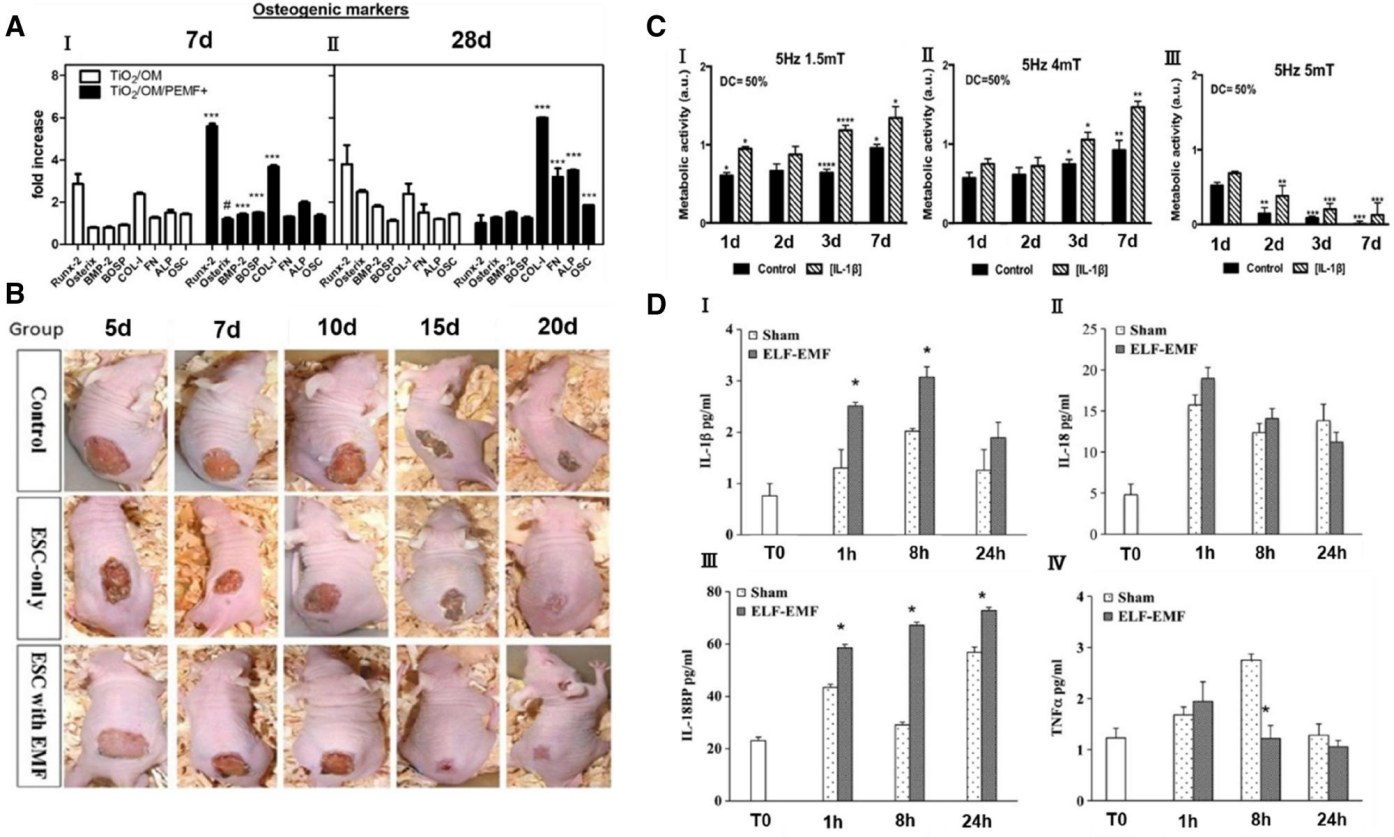
Biological effects of MFs in tissue engineering. (**A**) Differential expression of osteogenesis-specific genes in the presence and absence of magnetic stimulation. Reprinted with permission from Ref. [44]. Copyright 2018, Bloise *et al*. (**B**) Wound images of the control group, the ESC only group and the ESC with EMF group. Reprinted with permission from Ref. [45]. Copyright 2017, Wiley. (**C**) Effect of different MF strengths on human tendon-derived cells (hTDCs) treated with interleukin-1β (IL-1β). Reprinted with permission from Ref. [46]. Copyright 2019, Wiley. (**D**) Changes in the amount of cytokines released in the presence and absence of MFs. Reprinted with permission from Ref. [47]. Copyright 2018, Wiley.

According to the Food and Drug Administration, MFs with strength below 8 T have no significant impact on the physiological health of human being, thus people have used MFs with various strengths for the treatment of diseases. As shown in Figure 1A–D, low-intensity pulsed electromagnetic fields (EMFs) can effectively treat bone injuries [44], promote wound healing [45], tendon regeneration [46] and inflammation resolution [47]. It has been found that some excitable cells have significant response to static magnetic fields (SMFs) stimulation [48], while rotating magnetic fields (RMFs) have been shown to possess similar effects to SMFs. However, these stimulation methods are non-cell type specific and have limited spatial resolution (centimeter level) [49].

**Figure 2.**
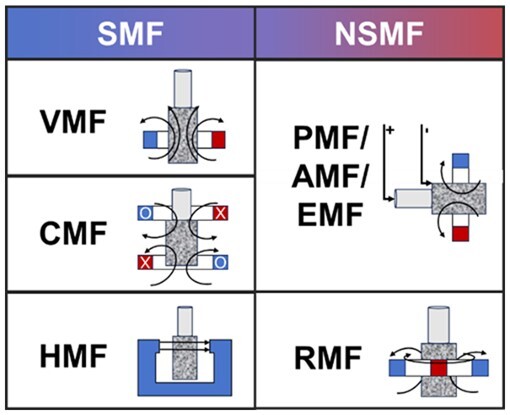
Classification of common MFs.

As shown in Figure 2, the common MFs include SMFs, pulsed magnetic fields (PMFs), RMFs, alternating magnetic fields (AMFs) and EMFs, which are briefly introduced as in the following sections.

### Static magnetic fields

A SMF is a MF with a fixed size and direction, usually generated by a steady current or permanent magnet [50]. According to the direction of MF and material action, it can be further subdivided into cusp magnetic fields (CMFs), vertical magnetic fields (VMFs) and horizontal magnetic fields (HMFs). SMF is generally considered safe for biological application because no current is induced by the Faraday effect [50]. In tissue engineering, the VMFs and HMFs generated by permanent magnets are more commonly used, while the CMFs are mostly used in smelting and forging. During the experiment, the required field strength magnitude and direction can be easily obtained by adjusting the relative position of the target to the field source.

### Pulsed and alternating magnetic fields

PMFs refer to MFs that does not change in direction but change in strength regularly, while AMFs refer to MFs that change in size and direction regularly. The EMFs generated by alternating current (AC) power supply are pulsating MFs, and the strength of the MF changes with the periodic changes of AC [51]. PMFs can be used to provide instantaneous local MF changes. The generation of PMFs can be caused by designing the circuit to apply a pulsed current to the inductive load [52]. Both PMFs and AMFs can exert oscillating forces on magnetic materials and can heat materials with high electrical conductivity, such as iron, copper and aluminum [53–55].

### Rotating magnetic field

A RMF refers to a MF in which the magnetic induction vector rotates in space with a certain regularity. RMF can also be generated by a variety of methods, such as by rotating a permanent magnet, the simulation models of which are typically RMF infinite and RMF φ-φ models [56, 57]. Under the action of the RMF, the magnetic material will be subjected to regular rotational force along the direction of the MF compared with the SMF. RMF has a large volume force, thus it is mostly used for magnetic stirring and particle acceleration [58, 59].

### Composite electromagnetic field

It has been proved that suitable electric and MFs can promote tissue regeneration [22, 23], and whether the two have a synergistic effect needs to be studied, thus composite EMFs have been developed. Composite EMF has MF and electric field at the same time, which can apply the composite stimulation of magnetic energy and electric energy to the material. The effect can be adjusted by changing the MF strength and current size, the structure of the composite field can also be designed and improved according to actual needs, thus showing a broader application prospect. Although it is currently less used in tissue engineering, EMFs have been shown to display beneficial effects on certain cells [60, 61].

## Magnetic materials

Materials that can react in a certain way to MFs are called magnetic materials. According to the strength of the magnetism of substances in the external MF, they can be divided into diamagnetic substances, paramagnetic substances, antiferromagnetic substances and ferromagnetic substances [62]. Common magnetic materials mainly include magnetic nanoparticles (MNPs), magnetic bioceramics, magnetic polymers and other magnetic response materials, while MNPs can be doped into the material structure by physical or chemical methods and prepared into various physical forms of magnetic tissue engineering scaffolds [63]. As shown in Table 1, magnetic materials have been extensively reported in the regeneration of tissues such as bone, muscle and nerve. Magnetic scaffolds can promote cell migration and differentiation by changing the microenvironment, magnetic particles encapsulated into cell through intracellular endocytosis could change cell gene expression, promote cell migration or adhesion, etc. [91–93]. Moreover, MNPs can be combined with external MFs to carry out remote operation of cells or intracellular biological macromolecules, drug delivery, disease diagnosis, etc. [91, 94–97].

**Table 1.** Application of magnetic materials in tissue engineering

Materials	Tissue	Effect	Ref
Magnetic silk fibroin protein	Bone	No toxic to osteogenic cells, improve cell adhesion and proliferation	[64]
Polymethyl methacrylate, drug-loaded magnetic phase change nanoparticles	Bone	Magnetic targeting, magnetic hyperthermia through minimally invasive injection	[65]
Superparamagnetic iron oxide nanoparticles (SPIONs), bioactive glass	Bone	Regenerate the damaged bone, magnetic hyperthermia	[66]
MgFe_2_O_4_	Bone	Improve MC3T3-E1 cells adhesion and proliferation, activate ALP, promote bone formation	[67]
SPIONs loaded into chitosan, collagen, hyaluronic acid and calcium phosphates	Bone	Improve cell viability, promote tissue regeneration	[68]
Fe_3_O_4_ nanoparticles, magnesium calcium phosphate bone cement	Bone, vascular	Promote the osteogenic differentiation and mineralization of BMSCs, facilitate angiogenesis	[69]
Magnetic cellular spheroids	Vascular	Assemble the cells and fuse into the vascular tissue structure	[70]
Magnetic bacterial cellulose	Vascular	Facilitate angiogenesis, prevent inflammation and thrombosis	[71]
RGD peptide-grafted magnetic bacterial cellulose	Vascular	Promotes cell adhesion and proliferation, regulate endothelialization	[72]
Magnetite cationic liposomes	Vascular	Prepare angiogenesis cell sheets, facilitate angiogenesis	[73]
Magnetic silk fibroin protein	Vascular	Excellent biocompatibility, promote the growth of vascular endothelial cells	[74]
Magnetic responsive hydrogel of methacrylated chondroitin sulfate	Tendon, bone	Affect cell morphology, promote tendon and bone regeneration gene expression	[75]
Magnetically responsive nanocomposite hydrogel	Tendon	Promote cell elongation and alignment	[76]
Hybrids of cellulose nanocrystals decorated with MNPs	Tendon	Promote cell alignment, promote tendon sheath production of HASCs	[77]
MNPs	Tendon	Stimulate the expression of tendon-related genes and protein synthesis	[78]
MNPs, starch and polycaprolactone	Tendon	Promote tendon componentization of ASCs under magnetic stimulation conditions	[79]
Magnetic spongy-like hydrogel	Tendon	Support human tendon-derived cell growth, adhesion, diffusion and migration	[80]
Biohybrid microswimmers	Muscle	Enable precise movement to a single C2C12-derived myotube, trigger myotube contraction	[81]
Remote magnetic short nanofibers	Muscle	Increase the arrangement of muscle fibers, enhance skeletal muscle regeneration	[82]
Alginate-coated magnetic microparticles	Muscle	Promote myoblast alignment	[83]
Magnetic bioreactor system, magnetic hydrogel	Muscle	Accelerate the differentiation of mouse myoblasts in hydrogels, increase the diameter and length of myotubes *in vitro*	[84]
Cobalt-based titanium dioxide (Co-doped TiO_2_) nanofibers	Muscle	Enhance cell adhesion and myoblasts growth	[85]
Magnetic nanochains	Nerve	Improve neural stem cells orientation, promote the directed growth of neurons	[86]
Mesoporous hollow Fe_3_O_4_ nanoparticles	Nerve	Induce polarization of macrophages, promote the proliferation of neural stem cells and the migration of vascular endothelial cells	[87]
SPIONs	Nerve	Induce and maintain the repair phenotype of Schwann cells	[88]
Self-rectifying magnetoelectric metamaterial	Nerve	Convert magnetic stimulation to electrical stimulation and wireless stimulation of peripheral nerves, restore sensory reflexes	[89]
Magnetic poly(lactic-co-glycolic acid) (PLGA) microcapsules	Nerve	Promote neurite extension, regulate the release of NGF	[90]

Magnetic materials have become a powerful tool for controlling receptor-specific signaling and controlling cellular behavior at the cellular and molecular levels [98, 99]. The magnetomechanical torque is proportional to the saturation magnetization strength (MS) and the applied MF strength (H) [100]. The magnetic particles must possess a high MS, achieved through careful design of their composition and geometry, in order to generate a mechanical force (>0.2 pN) [101] sufficient to initiate cell signaling processes. Additionally, it is essential for magnetic particles to exhibit negligible magnetization remnant (MR) in the absence of a MF in order to ensure their stability in water. Therefore, weakly ferromagnetic or superparamagnetic particles are considered to be suitable materials for biological applications [102, 103]. The coercive MFs of magnetic particles can be controlled by changing its composition, shape and magnetic crystal orientation [36]. MNPs with particle sizes smaller than 30 nm are magnetized only in the presence of external MF and are considered to be superparamagnetic [104]. Some of the materials commonly used to produce MNPs are iron, cobalt, and nickel, which may be toxic to cellular or *in vivo* applications [105]. The clinical use of Fe_3_O_4_ and γ-Fe_2_O_3_ has been approved, and they are considered to be biocompatible [106]. In addition, compared with magnetite, magnetite MNPs contain less oxidized iron (Fe^3+^) and show less damage to recipient cells [104] and the use of them has become more prevalent across a range of biomedical disciplines.

Due to the hydrophobicity and easy agglomeration of MNPs, they are usually coated with various biodegradable organic coatings to improve their dispersibility and endow them with the required functional groups [107, 108], among them, the modification of MNPs by dopamine treatment, silanization treatment, etc., have been widely used [109–113]. Modifying the surface of MNPs can improve its therapeutic effectiveness in tissue engineering and facilitate the precise delivery of drugs. Two primary approaches for the surface modification of MNPs are mainly employed: physical methods and chemical methods. Physical methods mainly rely on electrostatic interactions, hydrophilicity/hydrophobic interactions, affinity, etc., these methods have fast reaction speed and simple operation, but the binding to the MNPs is not strong enough, which is easy to cause leakage during drug delivery [114]. Chemical methodologies encompass the manipulation of MNPs’ surface through chemical reactions, facilitating the adhesion of pharmaceuticals or drug conveyors. Amongst these, a prevailing approach involves the incorporation of functional molecules, including drug substances, polymers or ligands, onto the MNPs’ surface through interaction forces between covalent or non-covalent bonds [115, 116]. Such chemical alteration permits steadfast drug encapsulation, regulated drug release, and can even bestow precise targeting capacity, fostering selective interaction with desired cells or tissues [117–119]. The drug targeting of MNPs is mainly achieved through the specific affinity of the drug or drug carrier surface for specific tissues and organs [120, 121] and the external physical signals of the specified target site, such as MF, temperature, etc. [122], while the release process of the drug depends on the sensitivity of the drug to certain physical stimuli, such as temperature [123, 124], pH [125], ultrasound, electric field, MF [126], light [127], etc. By changing these factors, controlled drug release can be achieved. The binding of MNPs to drugs can also extend the half-life of drugs, making drugs more efficient and active in their application [128, 129].

Magnetic materials have been used in various fields of tissue engineering, mainly in magnetic targeting, magnetic actuation, magnetocaloria and drug delivery [92, 130]. Currently, MNPs are the hot spot of research, and surface modifications are often required to improve the biocompatibility of MNPs. However, during the modification process, it is inevitable that some agglomerated particles will be coated, which will affect the uniformity of particle size, and some modification methods may reduce the magnetic responsiveness of MNPs [131, 132]. Besides, the accuracy of magnetic targeting still needs to be improved, and its short-term and long-term effects on normal tissue cells still need further research and clinical verification. In future research, in addition to focusing on the impact of magnetic materials on living organisms, we should also focus on the optimization of their composition and modification methods [90], in order to achieve cell-level targeting accuracy without affecting other cells, and precisely regulate cells through drug, mechanical and other effects. Moreover, electrical signals are also important regulatory signals for living organisms. The special properties and coupling behavior of magnetoelectric composites under thermal, electrical, magnetic and mechanical loads have also been widely studied [133]. How to reasonably design the composition and structure of magnetoelectric materials, and how to achieve the coordinated regulation of magnetic, thermal, electric and force on tissue regeneration, should also be a promising research direction.

## Effects of MFs on cells and tissues

Over the course of evolution, many organisms on Earth have developed the ability to sense the Earth’s MF [134]. The biological cascade of regulatory actions in cellular tissues provides a unique opportunity for magnetic applications. Especially in peripheral tissues, which are very challenging for implantable scaffolds due to the abundance of vascular and neural networks and the need to withstand forces or deformations on a regular basis [135], and thus wireless stimulation is considered to be very promising. However, electrical, optical and ultrasound stimulation are very limited due to their location and movement depth within the body, and magnetic stimulation has attracted much attention for its high penetration and low energy loss.

In mammalian cells, ferritin does not exhibit a significant magnetic moment of ferrous/ferromagnetism. Natural ferritin stores iron atoms in the form of hydrated iron, resulting in the formation of superparamagnetic nanoparticles with very weak magnetic moments [136]. MFs can exert temporary regulation over stem cell adhesion, differentiation and mechanosensing by triggering the release of bioactive factors through magnetic nanoswitches [137]. MF can also exert a repulsive force on diamagnetic compounds, which can cause physical deformation of the biomaterial matrix, and in turn stimulates various reactions in the cells [138]. High-frequency MFs have been shown to induce apoptosis and inhibit chondrocyte proliferation [139]. Besides, low-frequency MFs can control the orientation of magnetically labeled cells without observing any adverse effects on cell growth and proliferation [140–142]. Furthermore, MFs have the potential to modulate inflammatory responses and enhance the differentiation of M2 macrophages linked to inflammatory abatement [143]. Exposure to MFs has also been found to upregulate cell adhesion molecules in magnetically labeled mesenchymal stem cells (MSCs) [144, 145].

### Effects of SMFs on cells and tissues

**Figure 3.**
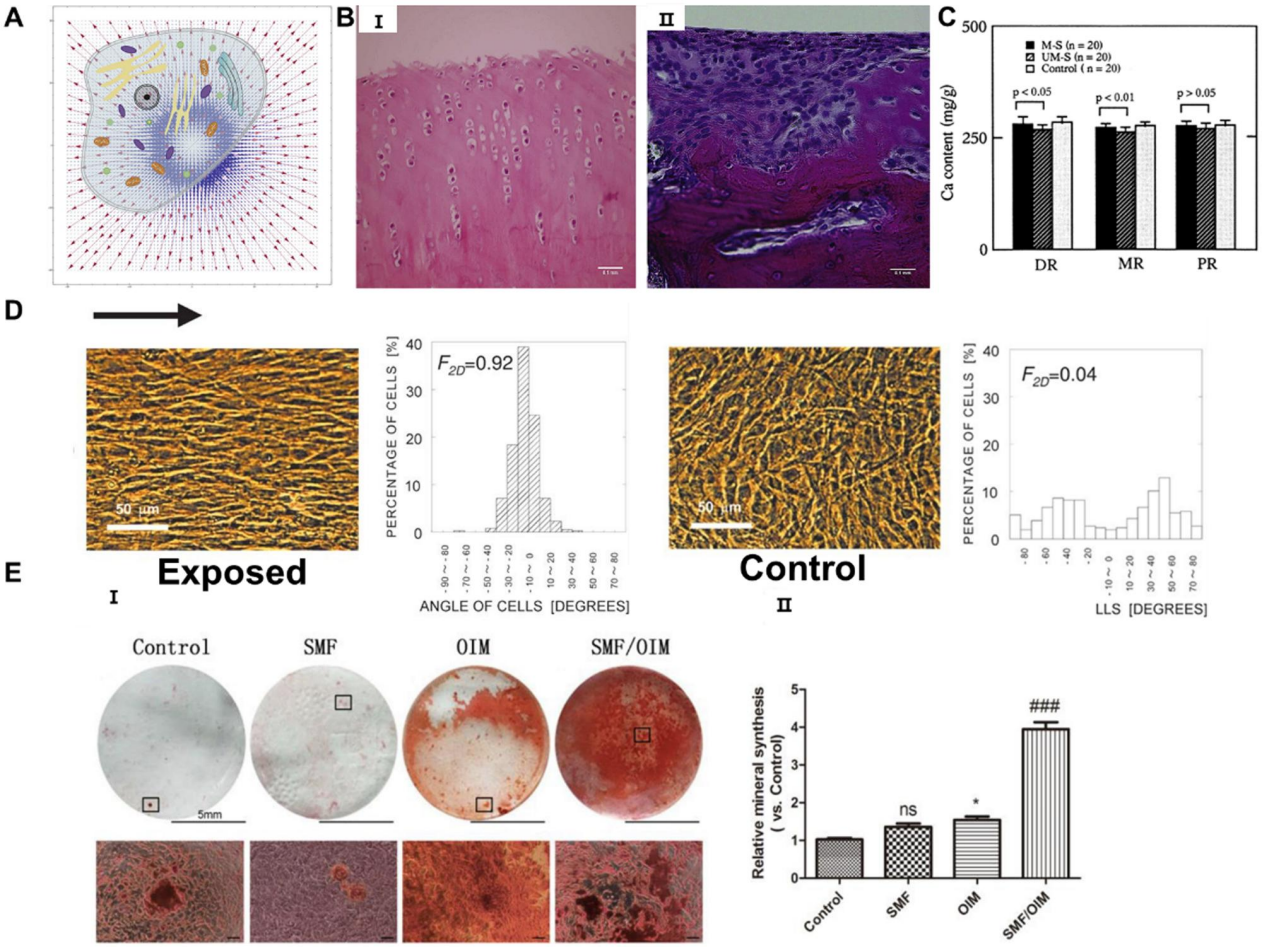
Effects of MFs on cells and tissues. (**A**) Cells in a non-uniform MF. Reprinted with permission from Ref. [146]. Copyright 2018, Wiley. (**B**) H&E stained images of kneecap cartilage defects in rabbits in the magnetic stimulation group (I) and control group (II). Bar represents 0.1 mm. Reprinted with permission from Ref. [140]. Copyright 2011, Elsevier. (**C**) Changes in femoral calcium content in rats with or without magnetic stimulation at 12 weeks postoperatively. Reprinted with permission from Ref. [147]. Copyright 1998, Elsevier. (**D**) Effect of SMF on MC3T3-E1 cells arrangement. Reprinted with permission from Ref. [148]. Copyright 2002, ASBMR. (**E**) (I) ARS staining of MCCs after 7 days of treatment. The scar bar = 100 μm. (Ⅱ) Evaluation of mineral synthesis. Reprinted with permission from Ref. [48]. Copyright 2020, Elsevier.

The cell is subjected to force in the MF (Figure 3A) [146]. When SMFs interacts with cells, magnetic interactions and free radical pair effects are generated, which ultimately lead to changes in cell biological behavior [149, 150]. The effects of SMFs on MSCs have been extensively studied (Table 2). 0.5 T SMF polarizes adipose-derived stem cells (ADSCs) and enhances intercellular interactions and mineralized nodule formation [159]. As shown in Figure 3B–E, SMF has long been found to promote the healing of radial fracture and repair of cartilage injury in rabbits [140], femur formation in rats and beagle dogs [147, 160], and oriented bone formation both *in vitro* and *in vivo* [148]. Li *et al.* [48] stimulated mandibular condylar chondrocytes (MCCs) with 280 mT of SMF and found that SMF accelerated osteogenesis by modulating the FLRT/BMP pathways. Yamamoto *et al.* [161] have demonstrated that SMF with magnetic strengths of 160, 280 or 340 mT had similar osteogenesis-promoting effects on rat cranial osteoblasts. This mechanism may be related to the activation of p38 phosphorylation at the cellular level for stimulating osteoblast differentiation [162]. Magnetic activation was able to initiate nuclear translocation of β-catenin to levels similar to Wnt3a, thereby enhancing proliferation and differentiation of skeletal progenitor cells and accelerating bone repair in Axin2 knockout mice [163]. By initially targeting the cell membrane receptor PDGFRα, higher mineral content appeared in the cells after culture in osteogenic medium for 3 weeks under magneto-mechanical stimulation [164]. All of the above studies suggest that the promotional effect of SMFs on osteogenesis may involve the combined effect of multiple substrates. It has also been reported that SMFs positively affect the stability of cell membranes by influencing their rotation through the antimagnetic properties of the phospholipids on the cell membrane, which leads to changes in cell shape, cytoskeletal rearrangements and ion channel function. Through these ion channel changes, SMFs can reduce intracellular calcium ion concentrations, which could be used to explain underlying mechanisms, including modulation of apoptosis, proliferation and cell viability [42, 165]. Besides, SMFs can also regulate blood movement in the skin through chronically altered vascular tone [166].

**Table 2.** Effects of different SMFs on MSCs

MSC origin	Nanoparticle	Magnetic device	SMF strength (mT)	Time of exposure	Effects of SMFs on MSC (compared to control groups)	Application	Refs
Human bone marrow	Ferucarbotran/Resovist^®^ (60 μg/ml)	Permanent magnet	600	24 h and 12 days	Reduction of colony-forming units, increased adipogenesis, and osteogenesis inhibition	Cartilages	[151]
Human bone marrow	Feridex (Tanabe Seiyaku)	Electro magnet	600	1 h	Increased expression of integrins and adhesion proteins	—	[152]
Murine bone marrow	None	Electro magnet	4, 7 and 15	1–4 days	Reduction of MSC viability and proliferation rates	—	[153]
Canine and equine adipose tissue	None	Permanent magnet	500	1–7 days	Increased MSC proliferation rates in both species; increased secretion of extracellular vesicles by equine MSCs	Bones and cartilages	[154]
Human bone marrow	None	Permanent magnet	400	14 days	Increased chondrogenesis	Cartilages	[155]
Equine adipose tissue	None	Permanent magnet	500	1–7 days	Ultrastructural changes; increased proliferation rate, colony-forming units, and secretion of extracellular vesicles; changes in vesicle content	Musculoskeletal and tendon	[156]
Human bone marrow	None	Permanent magnet	3, 15 and 50	1–9 days	Increased MSC proliferation rates; osteogenesis stimulation	Osseointegration between a dental implant and surrounding bone	[157]
Murine adipose tissue	Feridex (Berlex)	Permanent magnet	500	7 days	Reduction of MSC viability, proliferation rates, angiogenic cytokine release, osteogenesis and adipogenesis; phenotype shift	—	[158]

Reprinted with permission from Ref. [110]. Copyright 2017, Luisa H. A. Silva *et al.*

### Effects of PMFs on cells and tissues

PMFs are also widely used in tissue engineering. Sisken *et al.* [167] found that the stimulation of 4 h per day with 0.3 mT, 2 Hz MF significantly enhanced the regeneration of sciatic nerve defects in rats in 6 days. Byers *et al.* [168] found that the stimulation of 4 h per day with 0.4 mT, 120 Hz MF dramatically promoted the regeneration of facial nerves after 2 months. A low-frequency EMF of 2 Hz accelerated sensory nerve repair [169], while a variable MF of 6 Hz, 0.02 T might affect central nerve regeneration [170]. In addition, the very low-frequency MFs were found to induce stem cell differentiation for obtaining the desired phenotype [146, 171, 172]. Suszynski *et al.* [173] achieved effective nerve repair by stimulating with a high-field-strength (150–300 mT), low-frequency MF for only 20 min per day. PMFs increased blood flow in capillaries and the expression of serum copper blue protein, and cumulative treatment with PMFs promoted angiogenesis and indirectly the growth of blood vessels between nerve fibers, further providing sufficient nutrients for nerve regeneration [174]. Benedicta *et al.* [175] studied the positive effects of EMFs on myelin regeneration. The effects of EMFs on nerve cells may be related to ion transport, protein and growth factor metabolism. It has been reported that cells and various intracellular molecules respond to very low-frequency EMFs by increasing the intracellular calcium ion concentration [176]. Cho *et al.* [177] found that 50 Hz EMFs induced neural differentiation of bone marrow MSCs without the addition of differentiation factors. EMFs stimulation upregulated the expression of Cav-1 channels, thereby promoting neural stem cell differentiation [178]. PEMFs enhanced the regeneration of damaged tissues by transplanted cells, possibly due to intracellular metal ions variation. Liu *et al.* found that in PMF, an MF strength of 2.0 mT was suitable for the proliferation of Schwann cells (SCs). Meanwhile, the expression of some growth factors such as brain-derived neurotrophic factor, glial cell-derived neurotrophic factor and vascular endothelial cell growth factor was upregulated [179]. Glial cell-derived neurotrophic factors and vascular endothelial growth factor (VEGF) can stimulate neuronal proliferation and differentiation [180, 181]. Thus, PMF can improve SCs proliferation and thus promote nerve regeneration and recovery of related biological functions [182]. All of these results demonstrate that MF stimulation at a certain frequency and intensity can mitigate the adverse effects of nerve damage and accelerate nerve regeneration. In bone regeneration, PEMFs have been shown to promote fracture healing [183, 184], fusion of the spine [185], and growth of bone tissue into the interior of ceramic scaffolds [186]. Pooam *et al.* investigated the effects of PEMFs exposure on ROS-regulated gene expression by exposing HEK293 cell to a low-level MF. They proposed that PEMF exposure may transiently change SMF exposure conditions, thereby altering ROS synthesis in cell cultures, obtaining conclusions consistent with a free radical pair mechanism, and explaining how the redox chemistry of sensitive flavoprotein was manipulated by MFs, including geomagnetic fields [40].

### Effects of RMFs on cells and tissues

Generally, different strengths and frequencies of RMF elicit different cellular responses. Jedrzejczak-Silicka *et al.* found, by exposing HaCaT and L929 cells to different strengths and frequencies of RMF, an enhanced general metabolic activity was associated with the increased the level of ROS. However, the human keratinocytes stimulated by a higher frequency of RMF exhibited lower ROS and calcium ion concentrations, exhibited lower wound healing capacity, and thus low-frequency RMF may be beneficial for wound healing [187]. Besides, the low-frequency rotating magnetic fields (LFRMF) of moderate intensity have been shown to inhibit the growth of melanoma, hepatocellular carcinoma, mammary carcinoma and lung cancer in mice [188]. Exposure to RMF can ameliorate experimental autoimmune encephalomyelitis (EAE) by promoting the accumulation of CD4^+^ cells into peripheral lymphoid tissues, thereby improving the imbalance between Treg and Th1/Th17 cells [189]. Previous studies have found that the rotating non-uniform RMF exposure with 0.4 T exposure effectively promoted bone calcium content in thigh bones of ovariectomy rats and increased bone-specific alkaline phosphatase while decreased deoxypyridinoline cross-linking, confirming that strong MF exposure was effective in increasing bone mineral density and can be used to treat osteoporosis [190].

The effect of MFs on tissue and cells has been widely studied in the last several decades, but in recent years, new discoveries have been continuously explored. Various forms and intensities of MFs have been found to show beneficial or harmful effects on eukaryotes [191–193]. Its regulatory mechanism is very complex, mainly relying on magnetic induction proteins in organisms and some mechanically sensitive ion channels [194, 195]. Organisms such as pigeons and molecules such as ROS are regulated by MFs, but this regulation is specific. *In vivo* research experiments based on MFs almost always use remote magnetic stimulation *in vitro*. Although it is highly penetrating and does not require surgery, a large number of cells other than the target cells are also exposed to the MF environment, and the effect and safety of magnetic stimulation on other cells remain to be explored. The mode of action and field strength of the MF should be an urgent point to be solved in the future. And current studies of cell behavior under the influence of MFs still involve fewer cell types, mainly concentrated on some stem cells and nerve cells, etc. [196–199], thus the effect on other more cell categories needs to be further supplied.

## Effects of magnetic materials on cells and tissues

Due to the weak magnetization of ferritin in organisms, the conversion of MFs into various signals and the generation of more pronounced biological effects at the cellular and receptor scales are generally achieved through the synthesis of MNPs.

### Effects of MNPs on cells and tissues

**Figure 4.**
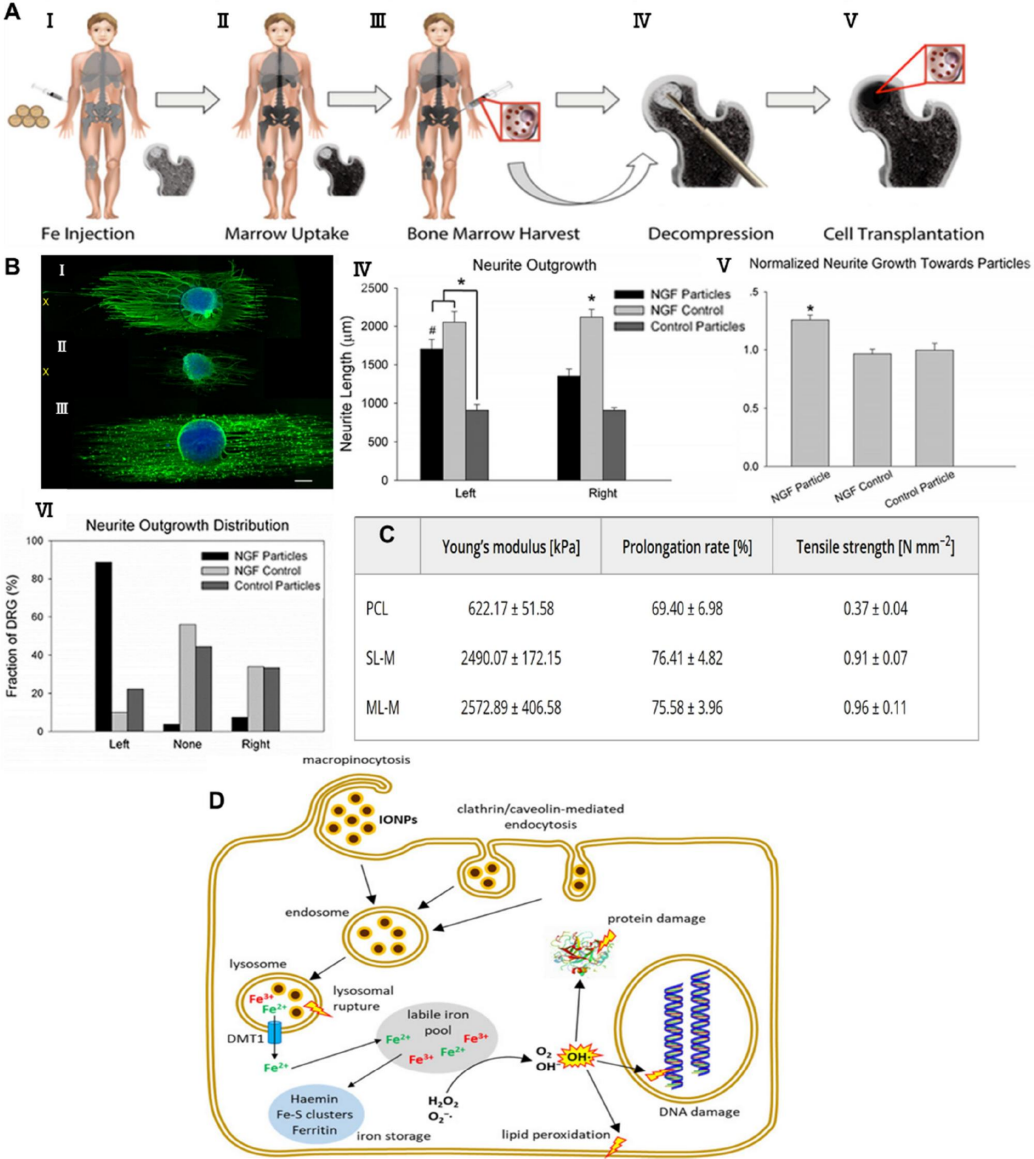
Effects of magnetic materials on cells and tissues. (**A**) With intravenous iron supplementation feroxytropol, cells in the normal bone marrow can be labeled and detected on MRI. Reprinted with permission from Ref. [204]. Copyright 2018, AACR. (**B**) The axonal orientation of DRGs cultured on PLLA fibers is preferentially guided by NGF-MNPs, and DRGs have longer axon lengths on the side with NGF-MNPs; scale bar= 500 μm. Reprinted with permission from Ref. [205]. Copyright 2015, American Chemical Society. (**C**) The mechanical properties of the scaffolds are enhanced after the addition of MNPs. Reprinted with permission from Ref. [206]. Copyright 2020, Wiley. (**D**) MNPs enter the lysosomal system of the cells through endocytosis. Iron can be released from MNPs and in turn induce a series of reactions. Reprinted with permission from Ref. [207]. Copyright 2021, Geppert and Himly.

Nanomaterials have been considered as a potential strategy to promote tissue regeneration due to their exceptional size, surface functionalization and chemical stability, as well as their electrical, magnetic and optical properties [200–202]. Among them, MNPs are frequently investigated due to their magnetoelectric properties and biological activity. MNPs themselves are equivalent to a magnetic domain, which can provide a MF at the nanoscale [203]. MNPs can be slowly deposited on the surface of cell membranes in the presence of a MF and bind to cellular receptors located on the surface of the cell membrane. As shown in Figure 4A, cells phagocytose the magnetic particles through endocytosis, making their entry into the cell more likely to affect the physiological functions of the cell [204]. MNPs can accelerate cell cycle progression by regulating the expression of cell cycle proteins and promote cell growth by decreasing intracellular H_2_O_2_ through intrinsic peroxidase-like activity [208]. When an MF is applied, MNPs are rapidly magnetized and generate mechanical forces that can be transmitted to the membrane to activate mechanosensitive ion channels [63]. Fe_2_O_3_ nanoparticles (γ-Fe_2_O_3_) with superparamagnetic properties have been reported to induce axon extension or direct protrusion growth under applied MFs without any side effects [205, 209] (Figure 4B). MNPs can induce autophagy in mouse dendritic cells, promote their maturation, and enhance therapeutic immune activation [210]. MNPs are also found to promote vascular endothelial cells survival from oxidative stress by enhancement of autophagy [211]. It has also been reported that MNPs could promote axonal outgrowth by activating mitogen-activated protein kinase signaling pathways [212]. And according to studies (Figure 4C), it has also been demonstrated that MNPs can be used to improve the mechanical properties of peripheral nerve scaffolds [, 213]. By coupling nerve growth factor (NGF) with iron oxide nanoparticles, the degradation of NGF can be significantly delayed, and even lower doses of NGF can achieve good therapeutic effects, promote the growth and differentiation of PC12 cells, which facilitates the growth of neural protrusions and increases the complexity of neuronal branching [128, 129]. *In vitro* studies, the toxicity of iron oxide nanoparticles on cells was experimentally found to be concentration- and size-dependent [214, 215], ranging from about tens to hundreds of μg/ml due to the surface properties. It has been reported that this toxicity is associated with oxidative stress and inflammation [207], and the mechanisms involved are shown in Figure 4D. Cells uptake MNPs into lysosomes through endocytosis, from which iron is subsequently released, and then the Fenton reaction and Haber–Weiss reaction occur. These reactions lead to the formation of hydroxyl radicals, which induce cell damage. Whereas its combination with melatonin has been shown to inhibit oxidative stress [206]. Kolosnjaj-Tabi *et al.* introduced iron oxide nanoparticles into rats by intravenous injection for a period of one year. They found that the particles were mainly concentrated in clusters in the lysosomes of the liver and spleen and that after a few months, the nonmagnetic iron pool increased with the disappearance of superparamagnetic iron, demonstrating the solubilized metabolism of the iron oxide nanoparticles in the lysosomes, showing their long-term biological nontoxicity [216]. Harrison *et al.* found that MSC, cardiomyocytes (CMC) and neural progenitor cells (ReN) showed a high dose of MNPs to be highly tolerance and showed effective MNPs uptake within 3 h, while cell viability was not affected by the process of MNPs uptake.

### Effects of magnetic composites on cells and tissues

It has been found that physical cues in the microenvironment of materials such as hardness, elasticity, topology, MFs, microcurrents, etc. can determine the fate of stem cell differentiation [208]. By adding MNPs to the material matrix, improvement of certain properties of the material and remote modulation of the internal structure of the material can be realized [217]. Li *et al.* prepared a single ADSC-scale substrate with a bi-isotropic structure assembled by 3D printing and magnetic-field-induced assembly of MNPs, and ADSCs cultured on the substrate with such a structure obtained a higher osteogenic rate, validating their conjecture that anisotropy at the cellular scale could better improve cellular sensing of the microenvironment. RNA-seq data showed that genes responding to the bi-isotropic structure were mainly enriched in cell adhesion, cytoskeletal and kinase signaling pathways, including the MAPK pathway and the PI3K-Akt pathway [218]. Omidinia-Arkoli *et al.* added SPIONs to PLGA solution for the preparation of staple fibers by electrostatic spinning/microdissection technique. The magnetic microfibers were then incorporated into a fibronectin-based hydrogel matrix and their orientation was achieved under a MF of 100–300 mT, depending on the length of the fibers and the concentration of SPIONs. The induced anisotropic organization of the microfibrils altered the mechanical properties of the hydrogel and also enabled remote control of the morphology and oriented growth of fibroblasts and neuronal cells [219]. Ganguly *et al.* prepared a magnetically oriented wound repair scaffold. The low-intensity MF treatment significantly enhanced the mechanical strength and anisotropy of the scaffold. And the scaffold promoted the growth of human skin fibroblasts, endothelial cells, and keratin-forming cells via the *in vitro* cell culture experiments, while *in vivo* it exhibited rapid wound healing with favorable results [220]. Johnson *et al.* mixed oleic acid-coated MNPs into electrospinning solution for orientation electrospinning. The electrospun fibers were crushed into short fibers and then mixed into the original solution of the hydrogel. Under the effect of MF, the rapid orientation of the fibers could be realized, and after cross-linking, a hydrogel containing oriented magnetic fibers could be further formed, which can be injected *in situ* into the injury site. In the *in vitro* experiments, the hydrogel significantly increased the length of DRG axons [221].

MNPs can also be used for remote manipulation of gel orientation. Antman-Passig *et al.* mixed MNPs into a collagen hydrogel and then applied an external MF on them. MNPs aggregated in clusters along the direction of the MF during the gel stage, leading to the orientation of collagen fibers. Neurons cultured in this hydrogel also formed an elongated directional arrangement with no effect on normal cellular activity [222]. Pesqueira *et al.* prepared a magnetic spongy hydrogel of protoelastin and found that the presence of MNPs altered the secondary structure of protoelastin. Morphologically, the hydrated protoelastin spongy hydrogel in the presence of MNPs showed significantly smaller pore size and less swelling compared to that without MNPs addition. Furthermore, *in vitro* biological studies using human tendon-derived cells demonstrated that magnetically responsive protoelastin spongy hydrogels supported cell survival and enabled cell adhesion, spreading and migration into the interior of the spongy hydrogels for up to two weeks [80]. Alginate gels containing MNPs were also found to promote the formation of capillaries from endothelial cells that provide nutrients to wounds and drain metabolic wastes [223]. Uto *et al.* prepared a PCL scaffold with nano-grooves, which showed good adhesion capability to human skin. Fibroblast culture on the scaffold showed that the scaffold promoted elongation and directed growth of fibroblasts. They also suggested the feasibility of integrating MNPs into the scaffold for remote manipulation in water [224]. In addition, a wound dressing was prepared by mixing MNPs and Ag nanoparticles into PCL for electrostatic spinning, which significantly improved the hydrophobicity of PCL. In *in vitro* cell culture, the survival of human skin melanocytes and the inhibition of Gram-negative *Escherichia coli* and Gram-positive *Staphylococcus aureus* were significantly enhanced with increasing Ag concentration. And in *in vivo* trauma experiments, the scaffold group was found to promote wound healing better compared to the control group [225].

### MNPs for cell labeling

Magnetic labeling with the appropriate density of magnetic particles has no detrimental effect on the safety and quality of cells [226]. Hu *et al.* [227] found that cells labeled with MNPs had enhanced vasculature generation function, which improved bone or cartilage regeneration. And when MNPs-labeled cells were preserved in cryogenic solution for 24 h under cryogenic conditions, Ren, MSC, and beating CMC cells maintained viability and differentiation potential [228, 229], and thus MNPs were used to control cellular functions and behaviors [230]. Intracellular delivery of MNPs allows cells to be localized in the presence of appropriate MFs and to form cell clusters that allow cells to be assembled into more complex tissue structures [231]. 3D bioprinting of cells can also be achieved by labeling cells with magnetic particles, which has great potential in seed cell culture. Vu *et al.* used a magnetic-based scaffold-free 3d bioprinting method for cell culture, which allowed the manipulation of cells to be assembled through the electrostatic interaction between magnetic particles and cells. Compared with traditional 3D cell culture using scaffolds, the protein content within the extracellular matrix of human skin fibroblast cells was significantly increased [232]. Based on this method, the efficiency of the analysis of the ECM proteome can be greatly facilitated and has the potential to be applied to skin trauma healing. Maria *et al.* similarly achieved the magnetic properties of human alveolar epithelial cells and human dermal fibroblasts by attaching polyelectrolyte-stabilized MNPs to their cell membranes, which conferred magnetic properties to the cells while avoiding potential cytotoxicity that could be brought about by internalizing the MNPs, and also achieved the controlled assembly of cells under MF manipulation [233].

Overall, the effects of magnetic materials on tissue and cells can be divided into two aspects: on the one hand, cells take up MNPs, MNPs and the bioactive factors attached to their surfaces directly affect the cell behavior, including growth, proliferation, stress resistance, etc. On the other hand, magnetic materials indirectly affect cell morphology, signaling, proliferation, and differentiation by affecting the environment around tissue and cells, including hardness, micromagnetic field, surface structure, etc. It should be noted that these influencing factors are related to the type, concentration, size and conditions of action of the magnetic material. When using magnetic materials for biological research, a thorough assessment of their effects on tissue cells is required to ensure safety and efficacy. And magnetic materials are often used in conjunction with MFs to achieve advanced functions such as targeting and assembly.

## Synergistic effects of MFs and magnetic materials on cell and tissue regeneration

**Figure 5.**
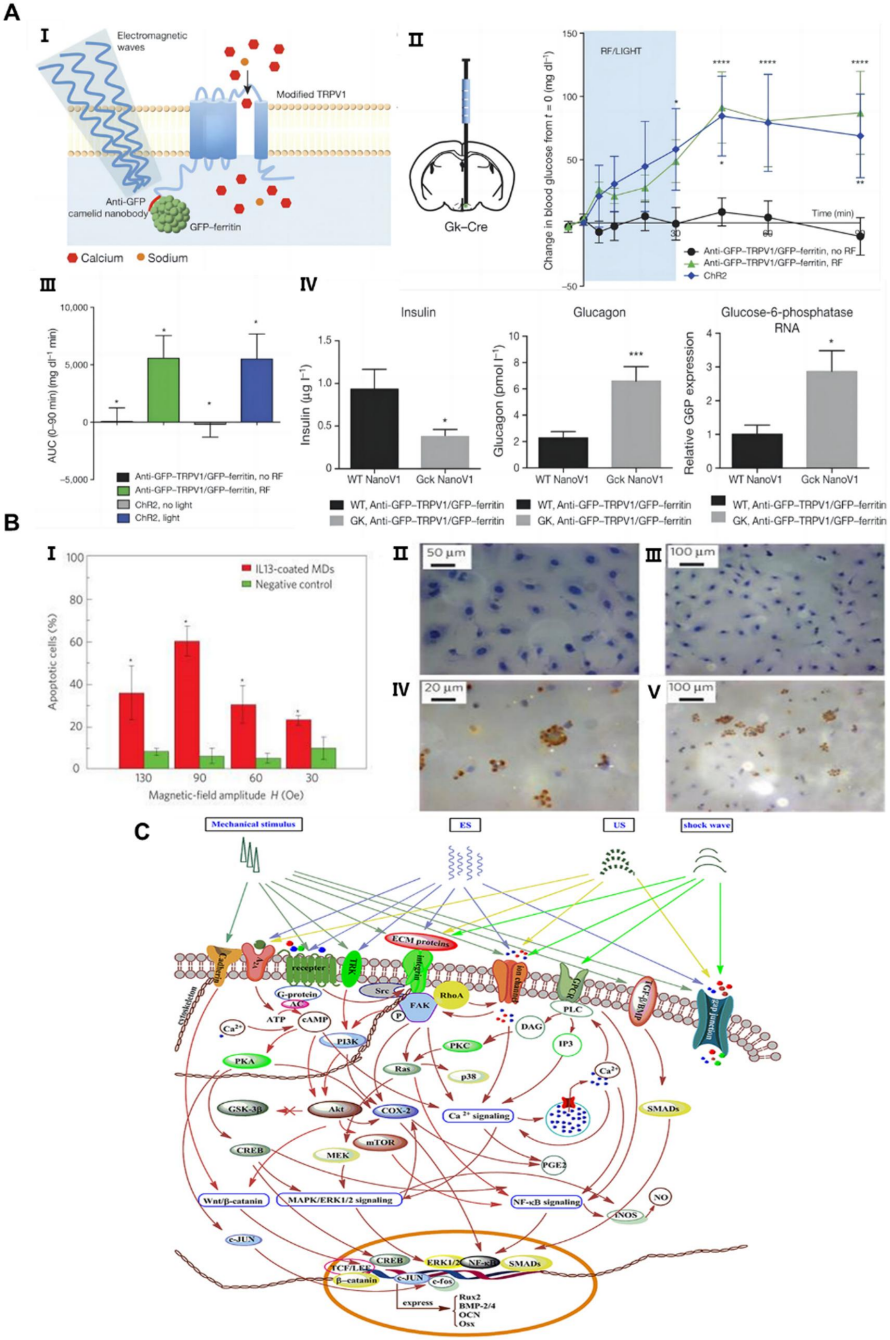
MFs and magnetic materials work together to regulate cell behavior. (**A**) Remote electromagnetic stimulation using radio waves activates nerves. (I) Diagram, (Ⅱ–IV) changes in blood glucose (Ⅱ) and cumulative blood glucose (III) and related gene expression levels (IV) with or without radio waves after injection of Ad-FLEX-anti-GFP-TRPV1/GFP-ferritin. Reprinted with permission from Ref. [238]. Copyright 2016, Nature. (**B**) MF triggers apoptosis through mechanical stimulation. (I) Effect of different MF strengths on apoptosis. (Ⅱ–Ⅴ) TUNEL staining images of negative control (Ⅱ, IV) and magnetic treated (III, V) cells. Reprinted with permission from Ref. [240]. Copyright 2010, Nature. (**C**) Possible mechanisms of osteocytes in response to mechanical stress. PI3K/Akt, Wnt/β-catenin, TGF-β/BMP, NF-κΒ, PKA, PKC, MAPK/ERK, Ca^2+^ and other signaling pathways are involved. Reprinted with permission from Ref. [241]. Copyright 2018, Springer.

Many cells in peripheral organs, including certain types of neurons, muscle cells and various endocrine cells have endogenous expression of thermal and mechanoreceptors [234, 235], and thus magneto-thermal or magneto-mechanical modulation through the combination of a MF and a magnetic material is considered to be a very feasible option [236, 237]. By implanting magnetic materials into specific sites or targeting specific types of cells, it is expected that precise modulation of the target of the movement state can be achieved by applying a MF that indiscriminately covers the entire site at the time of use, which can even be accurate to a specific receptor protein by adjusting the MF and magnetic material parameters. It has been reported that fusion of unmodified ferritin to the thermally and mechanically sensitive ion channels TRPV1 and TPRV4 is able to control neuronal activity and behavior using high-frequency AMFs and SMFs generated by permanent magnets [99, 238, 239], as shown in Figure 5A. In addition, many cellular structures, such as the cytoskeleton anchored to membranes and adhesion proteins, respond to mechanical forces [242]. It has also been shown that increased cell and tissue growth can occur in response to mechanical stresses generated by surface matrices or fluid flow [243], thus small deformations generated by tissue-engineered scaffolds in response to MFs can also exert a responsive effect on cell growth. In magnetomechanical modulation, a SMF and its gradient act on the magnetic moment of a nanoparticle to apply force or torque on the target. This method has been used to study cellular and macromolecular mechanics using magnetic tweezers [240] (Figure 5B), to disrupt tumor cells and modulate cell signaling [240, 244, 245]. In these studies, MNP composites with zero net magnetic moment in the absence of a MF produce a large magnetic moment in the presence of a weak MF (≤50 mT).

Forces in the range of 0.2–50 pN can trigger the cell's mechanosensitive receptors without causing damage to cell function [101]. This force can be calculated analytically or measured with an instrument such as an atomic force microscope [240, 246]. Immunohistochemistry, marker assays and behavioral tests can be used to confirm *in vivo* magnetomechanical regulation. In addition, the distribution of MNPs in cells can be visually observed by transmission electron microscopy or by fluorescence dye labeling and microscopic imaging. The resulting biological effects can also be further analyzed through tissue staining, behavioral and electrophysiological experiments [247–250].

**Figure 6.**
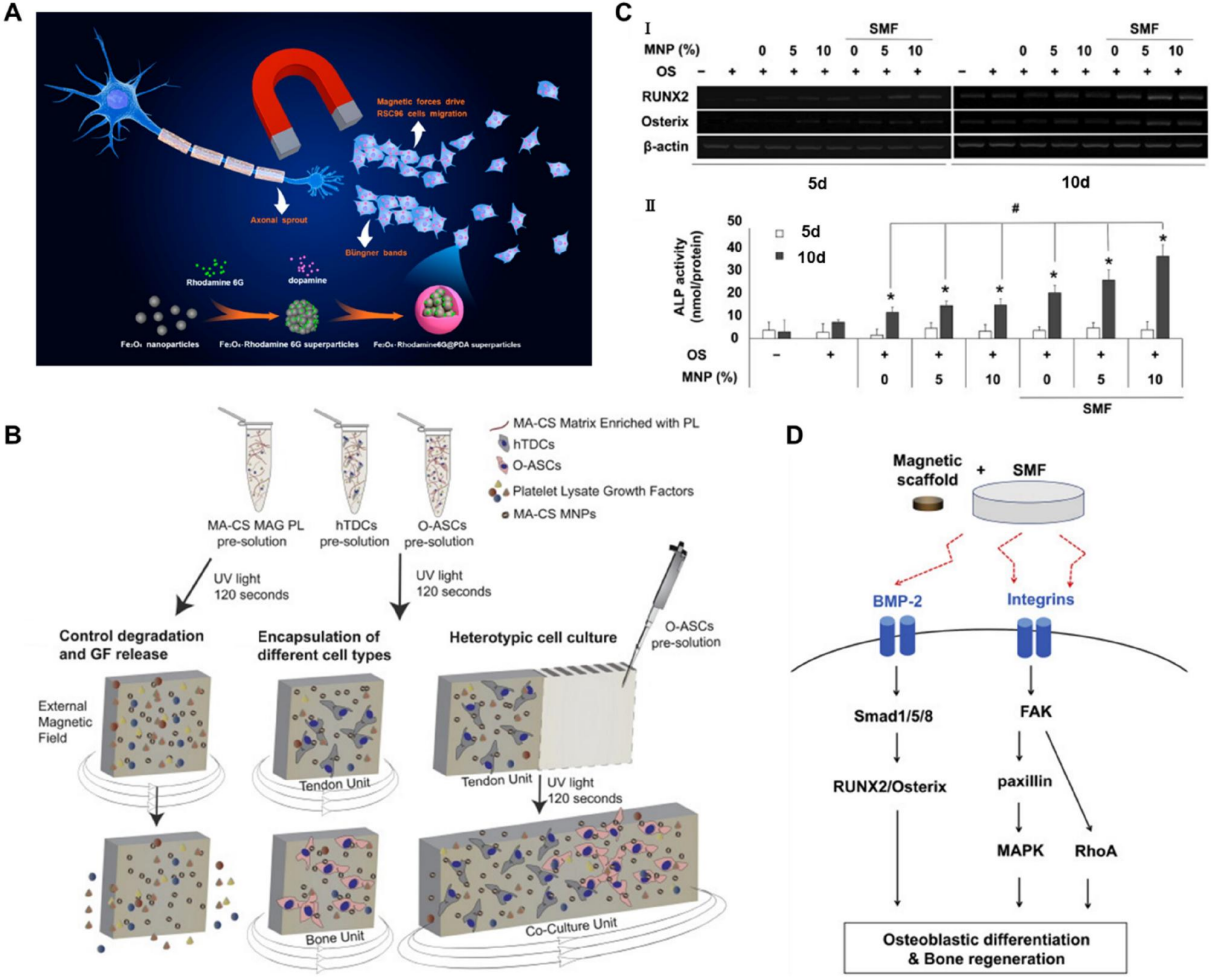
Synergistic effects of MFs and magnetic materials on tissue regeneration. **(**A) Mechanism of cell migration driven by MF and MNPs. Reprinted with permission from Ref. [132]. Copyright 2020, ACS Publications. (B) Schematic diagram of drug release and cell culture in a magnetic hydrogel. Reprinted with permission from Ref. [75]. Copyright 2018, Elsevier. (**C**) Effect of MF versus magnetic scaffold on osteoblast differentiation. Reprinted with permission from Ref. [258]. Copyright 2016, Elsevier. (**D**) MF and magnetic scaffold activate subsequent pathways by stimulating integrins, BMP-2 proteins in osteoblasts, ultimately promoting bone regeneration. Reprinted with permission from Ref. [258]. Copyright 2016, Elsevier.

The physical properties of biomaterial scaffolds are key cues that can activate intracellular biochemical signals [251], and physical stimuli, such as MFs, can also have a significant impact on cell fate and behavior by modulating various intracellular signaling pathways [241] as shown in Figure 5C. Magnetic materials synergistic with MFs thus have the potential to enhance cellular behavior by internalizing in cells to produce certain biochemical effects or by remote manipulation with external MFs to transmit mechanical signals that promote the activation of signaling pathways related to cell proliferation, migration and differentiation [252–257]. Ana *et al.* evaluated the biological properties and functions of cellulose nanofiber scaffolds decorated with MNPs using cultured human adipose stem cells (HASCs) with or without magnetic actuation. It was shown that magnetomechanical stimulation promoted highly organized cytoskeletal anisotropy and directed the mechanosensitive YAP/TAZ signaling pathway [77]. Liu *et al.* loaded SCs onto magnetic nanocomposite scaffolds consisting of MNPs and chitosan-glycerophosphate, and applied an external MF to repair sciatic nerve defects in rats. They found that the effect of external MF could significantly increase the vitality of SCs on the scaffolds, MNPs could play a certain cross-linking effect on the polymers, leading to a more compact internal structure. Compared with the application of magnetic scaffolds alone, the combined application of magnetic scaffolds and MF could greatly increase the number of regenerated myelinated axons, regenerate more neurons, and achieve a better repair effect [213]. SCs play a key role as neuroglia in the process of axon regeneration. The orderly migration of SCs facilitates the connection of extracellular matrix and the formation of Büngner’s band, which greatly promotes axon regeneration by providing mechanical support and secreting growth factors. The team of Wang designed and characterized a novel fluorescent-magnetic bifunctional Fe_3_O_4_-rhodamine 6G @ poly (dopamine) superparticles [132] (Figure 6A), and investigated in detail the effects and target migration mechanisms produced by SCs after ingesting the magnetic particles. They found that cells could sense external magnetomechanical forces and transduce them into intracellular biochemical signals that stimulate the expression of genes associated with SCs migration [132]. Silva *et al.* [75] integrated MA-CS MNPs into MA-CS hydrogels, and by applying an EMF, making it possible to externally manipulate the system and control the intrinsic properties of the constructs, including regulating the release of growth factors (Figure 6B). Yun *et al.* [258] demonstrated that 15 mT SMF synergized with magnetic scaffolds could upregulate the expression of osteogenic markers such as alkaline phosphatase (ALP) (Figure 6C) and activate endothelial cell vascular differentiation, and the related mechanism is shown in Figure 6D. Besides, the superparamagnetic nanoparticles were demonstrated to be able to support cell adhesion and proliferation under the influence of a weak MF by incorporating superparamagnetic nanoparticles into electrostatically spun HA/polylactic acid scaffolds (5–25 mT) to support cell attachment and proliferation, and the scaffold induced osteocalcin-positive cells earlier and in greater numbers in the presence of an external MF, leading to faster bone formation in bone defects [259].

The combination of MF and magnetic materials is extremely extensive, which can not only realize the long-range high penetration of MF, but also achieve specific targeting effects and improve operation accuracy. Some magnetic materials produce mechanical effects under the action of an applied MF, such as MNPs under an applied MF. The force will have an impact on the structure and function of biomolecules within the cell, and may affect the rearrangement of the cytoskeleton and cell movement, among others. Under the action of AMFs, certain superparamagnetic materials (such as MNPs) can produce thermal effects through magnetic loss [260], which in turn affect cell activity, metabolism and apoptosis. In addition, when the magnetic material is subjected to the gradient MF, it may also produce a local force gradient, which further affects the arrangement, differentiation and migration of surrounding cells together with the magnetic gradient. The joint application of MF and magnetic materials provides new means and possibilities for tissue engineering research, including 3D cell printing, magnetic nanorobots, construction of multifunctional magnetic materials, targeted controlled-release drugs in the future, with broad development prospects. When a MF is applied, it often brings complex stimuli such as mechanical stimulation, thermal stimulation and magnetic stimulation. The mechanism by which various stimuli interact with each other is difficult to fully elucidate. In addition, the surface properties of the material also have a non-negligible effect on the surrounding cells. There is still a huge gap to be filled in this field, which may also be a future research direction.

## Conclusion and perspective

The potential of magnetic strategies in tissue regeneration is being increasingly recognized through numerous studies. Modulation of cellular functions through magnetic materials activates important pathways involved in tissue regeneration and provides directional guidance for tissue regeneration, which is an advantage over other strategies. Therefore, further research is needed to design magnetic materials that can be precisely controlled to differentiate between treating healthy and affected tissues, however current magnetic stimulation protocols struggle to effectively differentiate between injured tissues for precise stimulation. New computational models and measurement methods are expected to achieve full-time and spatial recording in the operation process, and the rise of artificial intelligence also provides new ideas for the regulation of MFs. The further development and application of miniature magnetic robots is also expected to achieve molecular-level manipulation *in vivo*. Another major limitation related to the application of magnetic materials is the lack of biodegradability of conventional magnetic materials (iron, nickel, cobalt, etc.), which limits their potential application in regenerative medicine, and usually needs to be combined with degradable biomaterials in order to change the chemical properties of the material, but at the same time, the magnetic responsiveness of the material may be compromised. Rapid clearance of MNPs is also crucial after their role in the organism. Their prolonged stay may trigger a range of unintended reactions. In order to facilitate clinical applications, further reducing the size of MNPs or exploring new magnetic nanomaterials that are easily degradable may be potential solutions.

Current applications of magnetism extend beyond the use of MFs or materials alone, as they are now being combined with drugs and stem cells. The advent of novel detection and characterization techniques may inspire new designs and applications. Magnetogenetics is an emerging field that aims to use MFs to precisely reprogram cellular functions in spatiotemporal mode. Further investigation into the physical limitations of magnetogenetics and the underlying mechanisms of physiological changes observed with iron-binding protein structures could provide new insights for magnetic regulation. However, the current understanding of these mechanisms remains limited and requires further research. The effects of temporally or spatially alternating MFs on tissues and cells have not been thoroughly investigated, and there is a need to improve the generalization or targeting of different species and the specificity of targeting specific cells or molecules. In addition, magnetic manipulation of cells or molecules is currently mostly limited to a single magnetic material. By changing the composition or particle size to obtain MNPs with different magnetic responsiveness, and labeling different cells or peptides in a non-uniform MF, it is expected that more precise assembly of cells and even biological macromolecules can be realized with the further improvement of MF accuracy.

In clinical therapy, magnetic materials are widely used in the fields of bioimaging, magneto-thermal therapy and magnetic target guidance, but the design requirements of magnetic materials vary greatly from one treatment strategy to another. In the magnetothermal therapy system used for tumor tissues, it is necessary to focus on the limitations of magnetic materials such as the magneto-thermal conversion efficiency and the depth of tissue penetration. When targeted drug delivery is required, magnetic materials need to have a large specific surface area and ligand coupling properties, and can also work synergistically with other smart-responsive materials, such as thermosensitive polymers that can be magneto-thermally affected for drug release. In cases of nerve or bone injuries, which often require surgical implantation of materials, minimizing the incision to achieve the desired recovery effect is an important area of study. Researchers are exploring *in situ* injection materials that can effectively fill small incisions and conform to the diverse shapes of damaged areas.

Current research has focused on a few common signaling pathways, but in fact, many others remain to be explored. Combining magnetic materials to achieve remote activation of cellular signaling pathways holds considerable promise. While current studies have not identified significant toxic effects of magnetic materials and MFs on tissue cells, these studies have been conducted over relatively short periods of time. The long-term effects on organisms remain largely unexplored. Future studies should further strengthen the joint application of MFs, materials, stem cells, growth factor and drugs in both temporal and spatial dimensions to elucidate the underlying mechanisms. All the above knowledge will form the basis for further design efforts aimed at achieving the goal of fully regenerating new organs.

## Supplementary Material

rbae048_Supplementary_Data

## Data Availability

All data generated or analyzed in this study are included in published articles and their supplementary material documents and are obtained from the respective authors at reasonable request.
