# Supplementary material for: Application of magnetism in tissue regeneration: recent progress and future prospects
Source: Regen Biomater. 2024 May 7;11:rbae048. doi: 10.1093/rb/rbae048 (PMC11208728; doi:10.1093/rb/rbae048)

**Supplementary informations**

**Application of Magnetism in Tissue Regeneration: Recent progress and Future Prospects**

Wenchao Guan^a#^, Hongxia Gao^a#^, Yaqiong Liu^a^, Shaolan Sun^a^, Guicai Li^a,b*^

^a^ Key laboratory of Neuroregeneration, Co-innovation Center of Neuroregeneration, Nantong University, Nantong 226001, China

^b^ State Key Laboratory of Polymer Materials Engineering, Sichuan University, 610065, Chengdu, PR China

#These authors contributed equally to the work

E-mail：gcli1981@ntu.edu.cn

**All referenced figures and table in this article have obtained copyright permission, as listed below:**

**Figure 1A**


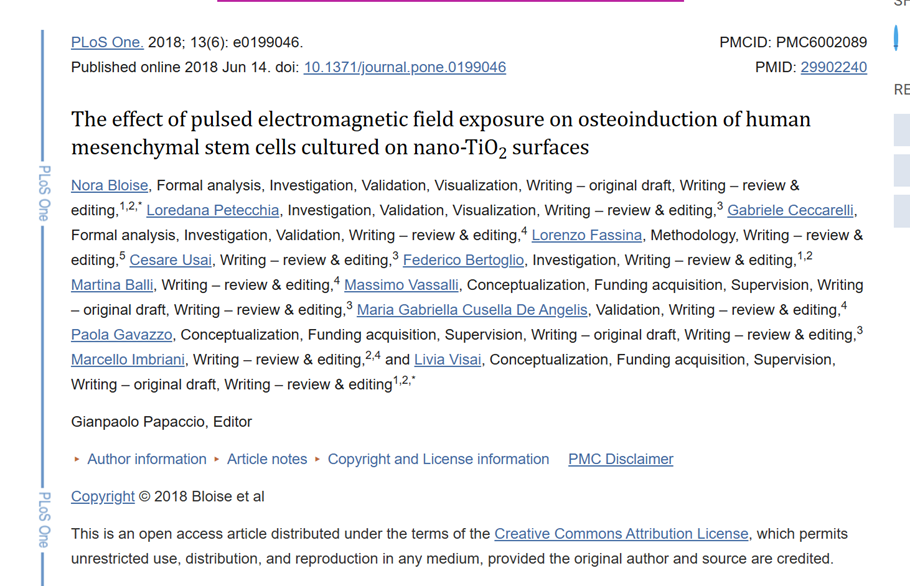


**Figure 1B**


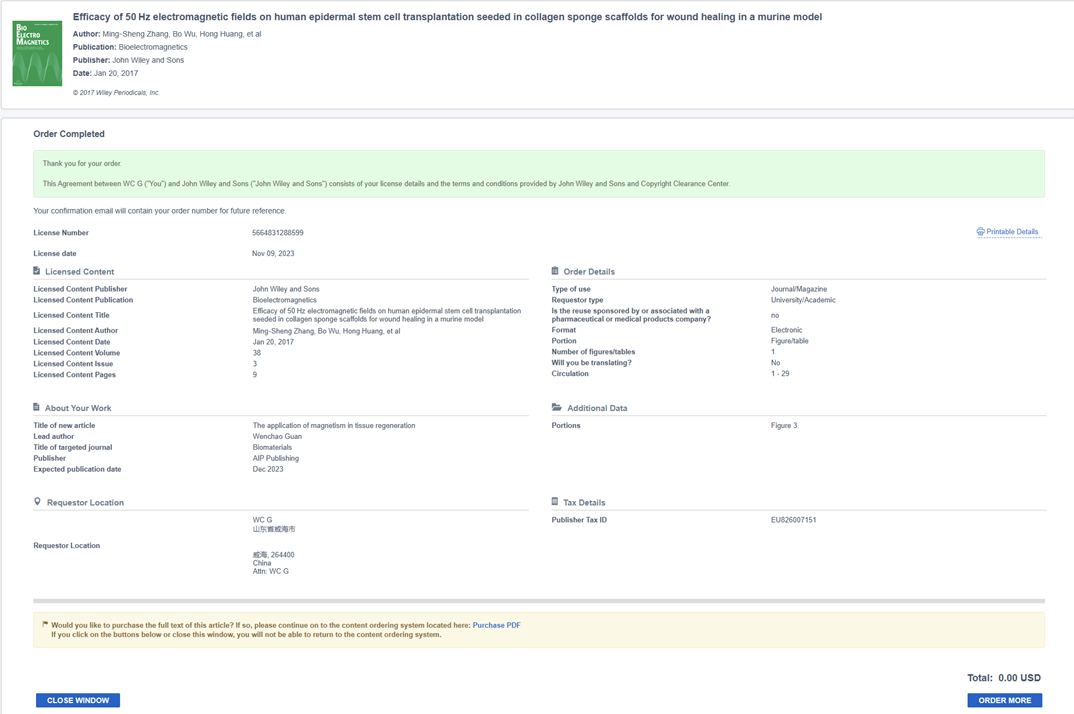


**Figure 1C**


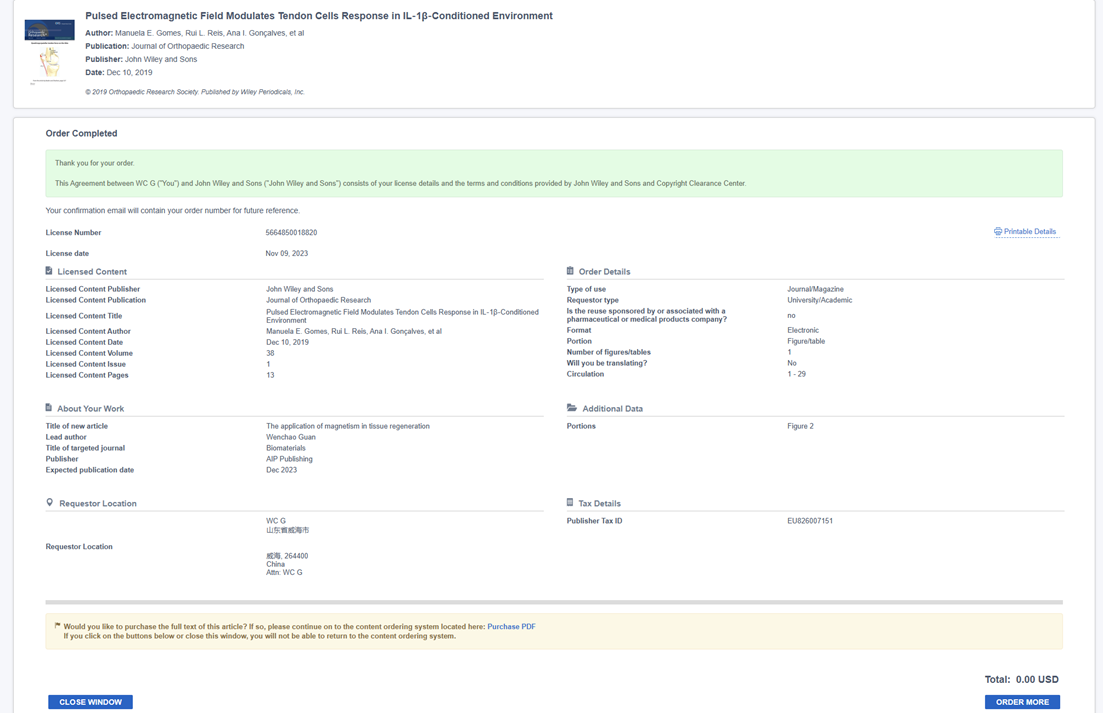


**Figure 1D**


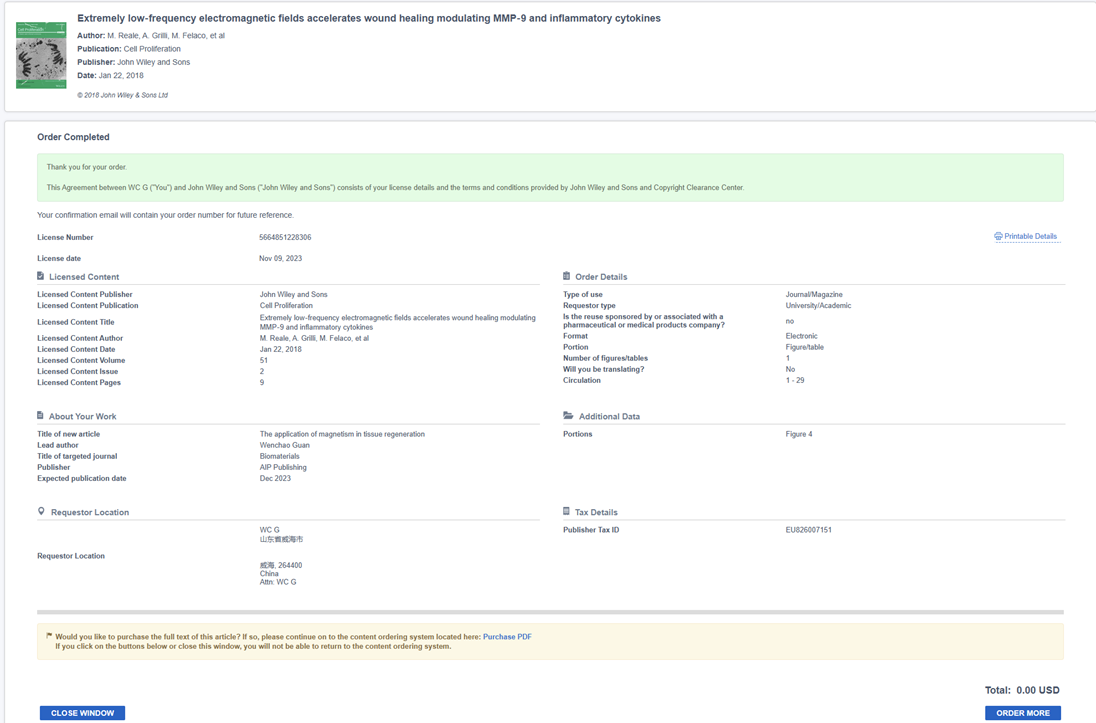


**Figure 3A**


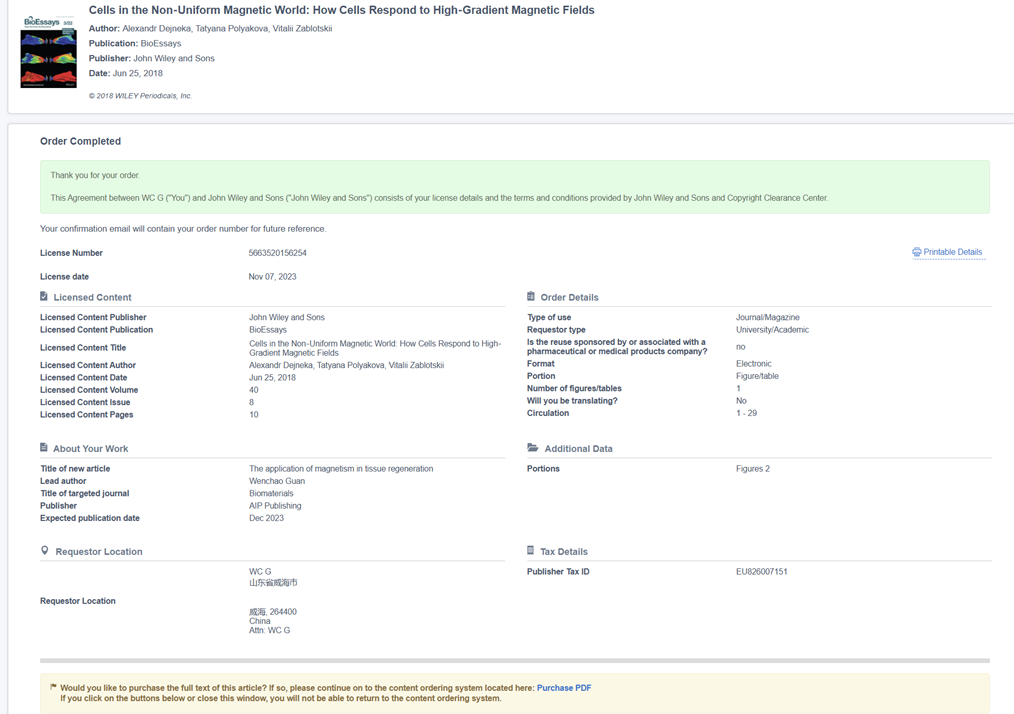


**Figure 3B**


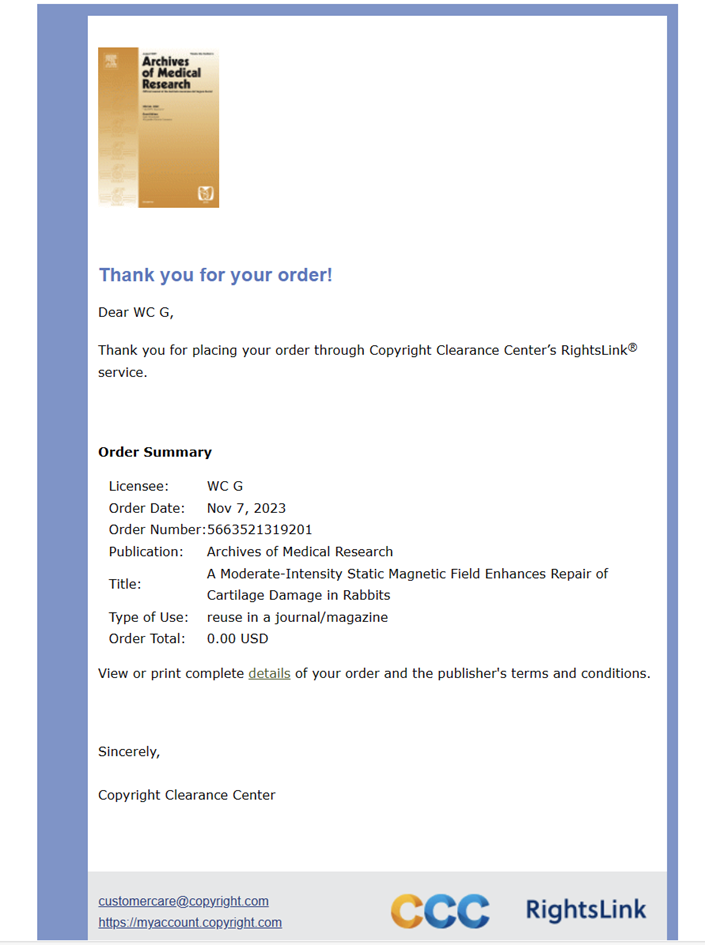


**Figure 3C**


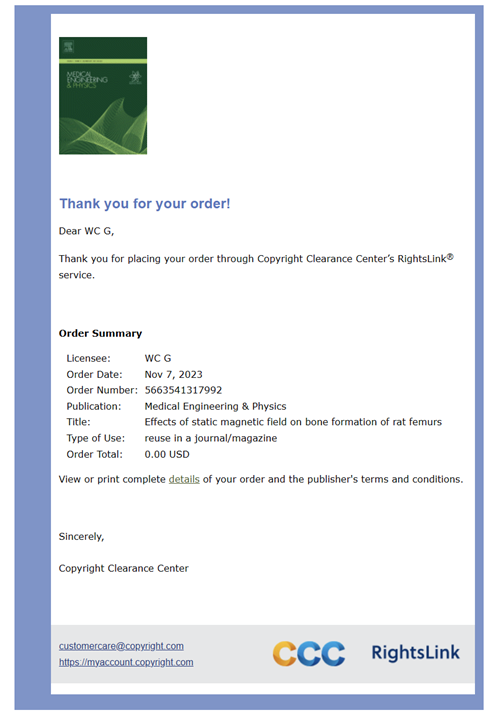


**Figure 3D**


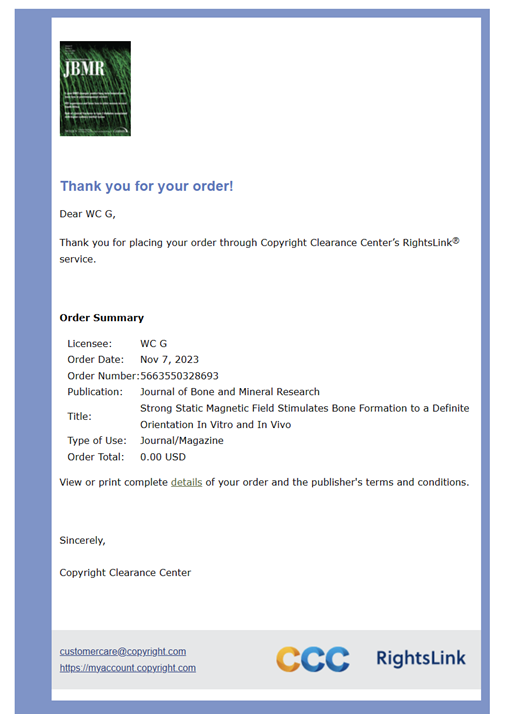


**Figure 3E**


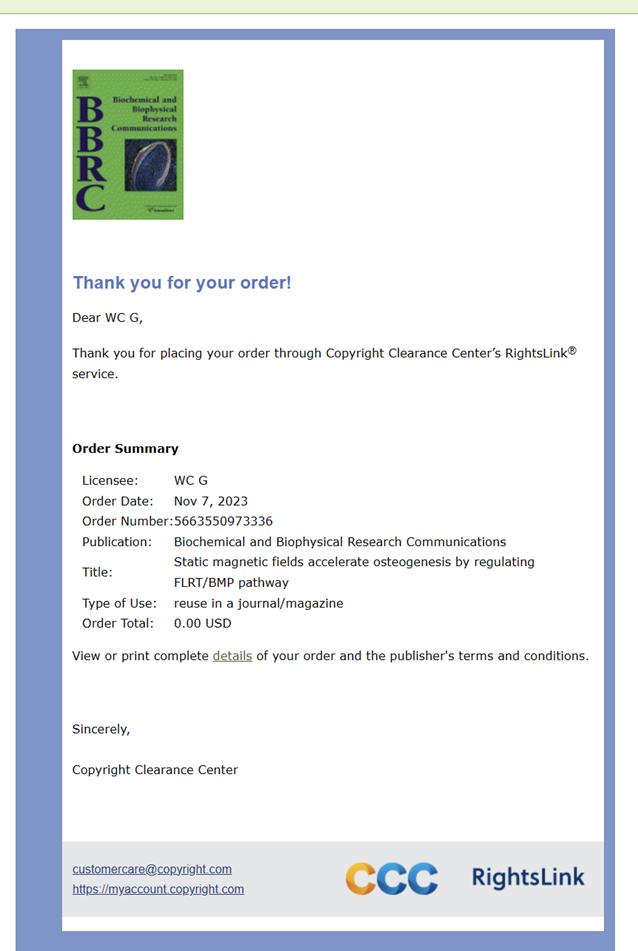


**Figure 4A**


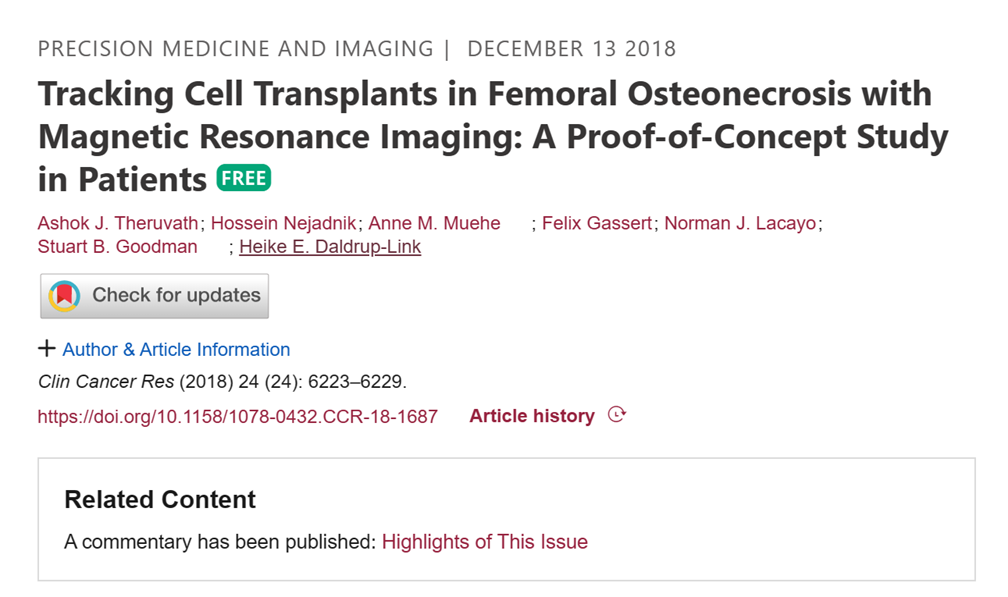


**Figure 4B**


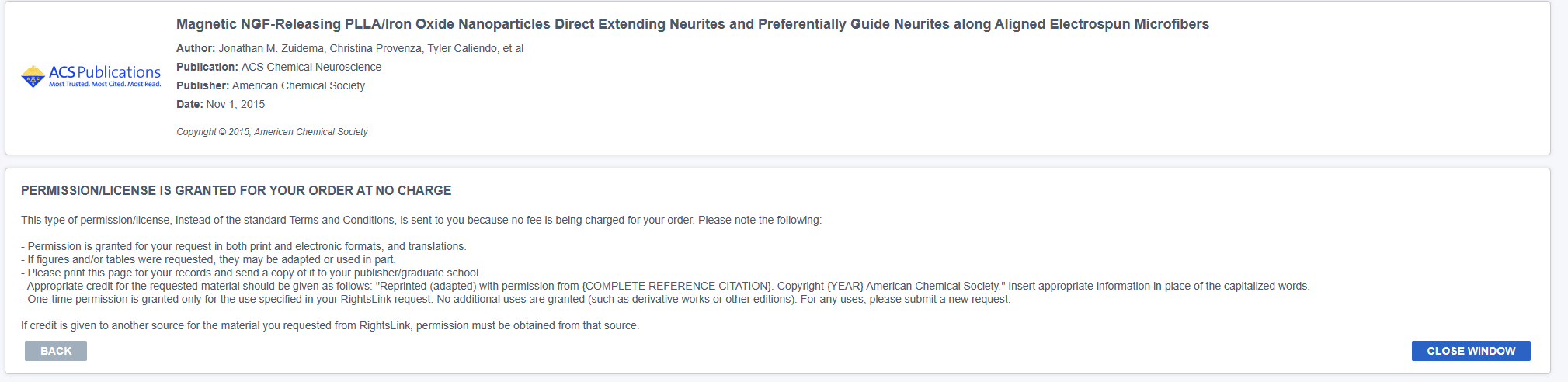


**Figure 4C**


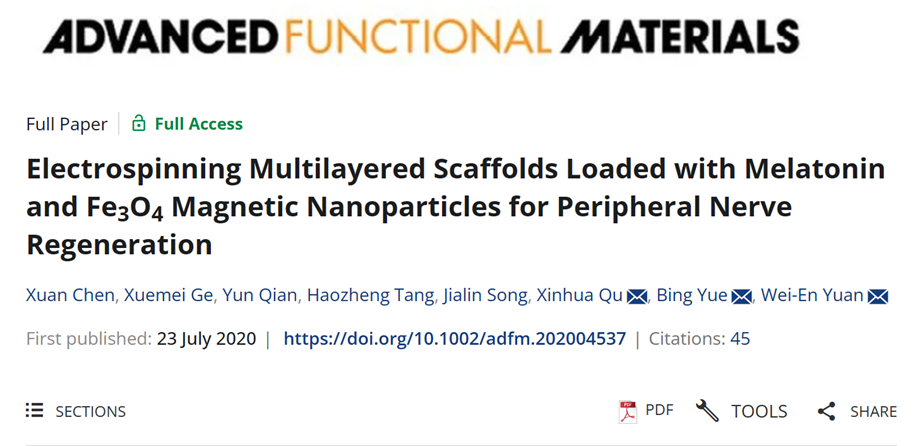


**Figure 4D**


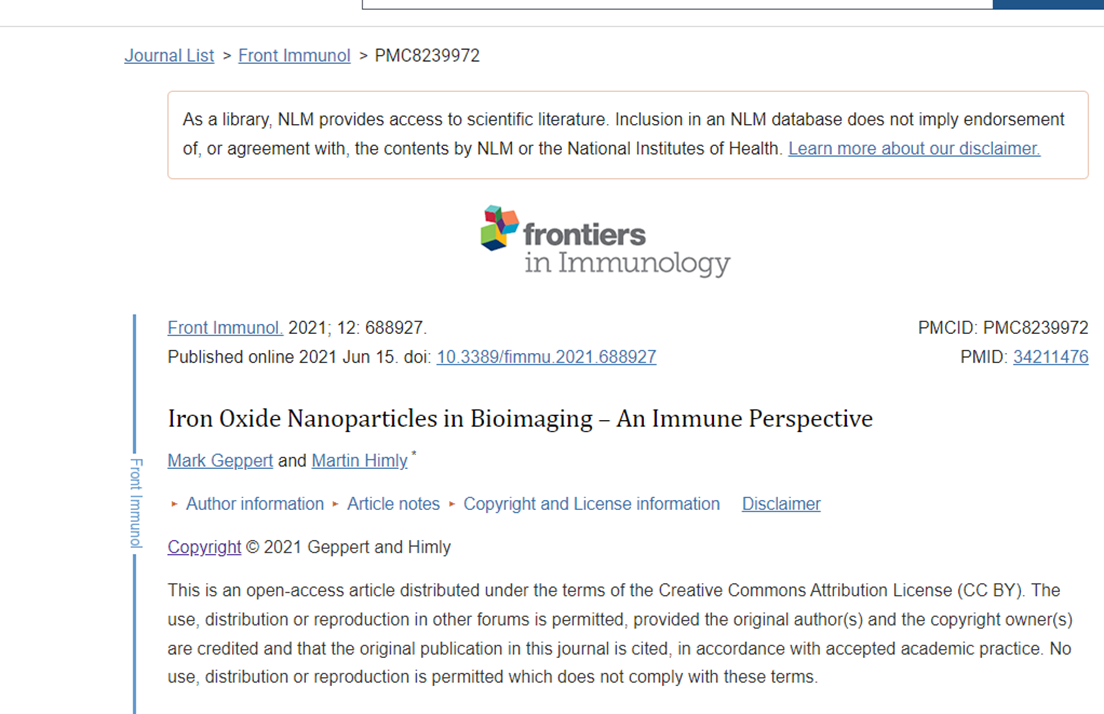


**Figure 5A**


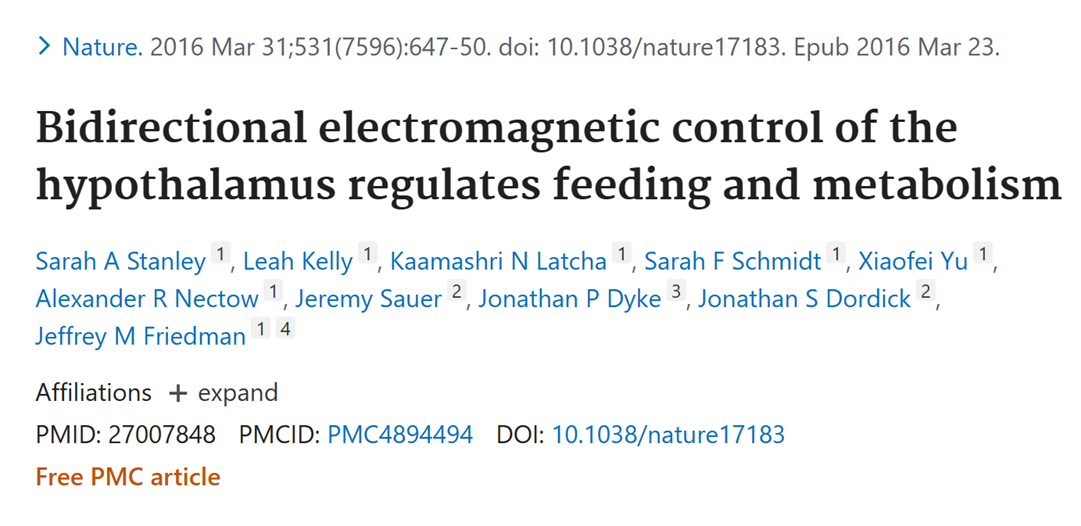


**Figure 5B**


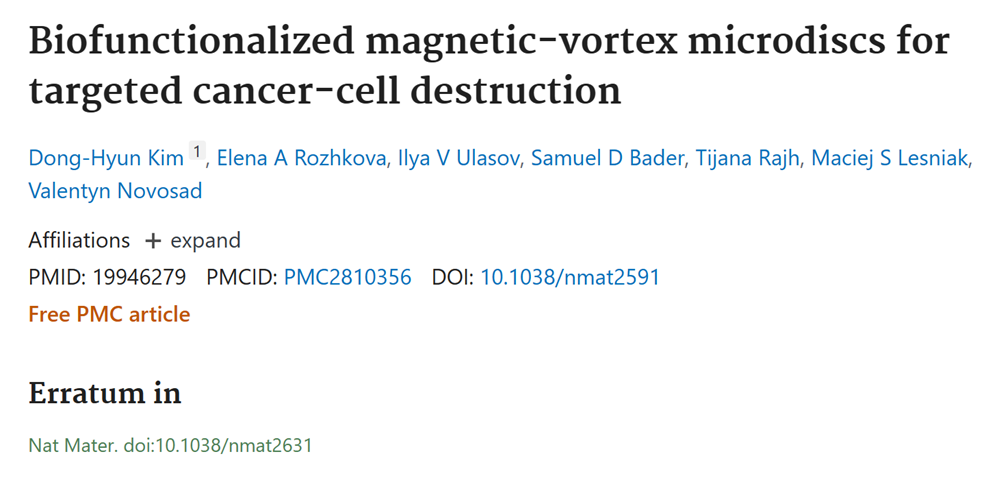


**Figure 5C**


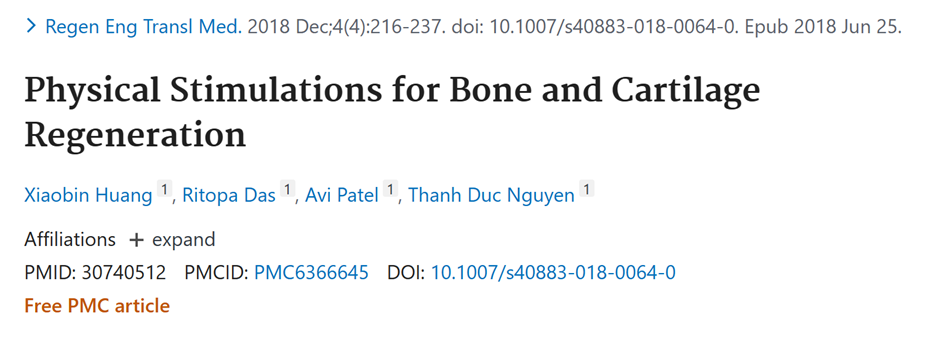


**Figure 6A/B**


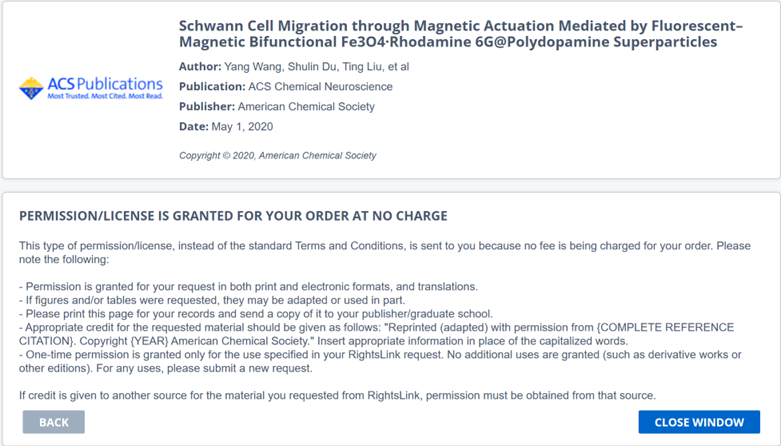


**Figure 6 C/D**


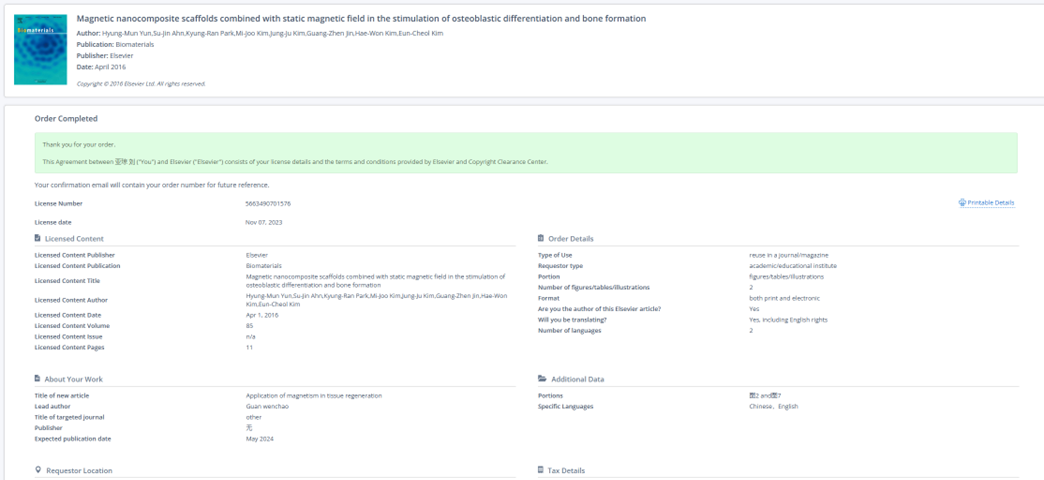


**Table 1.**


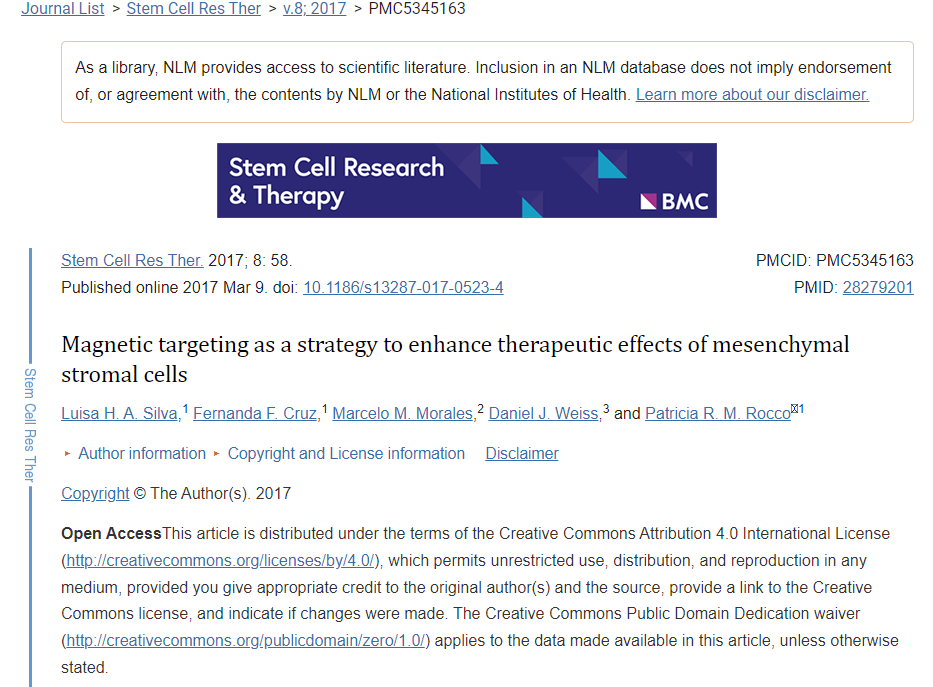


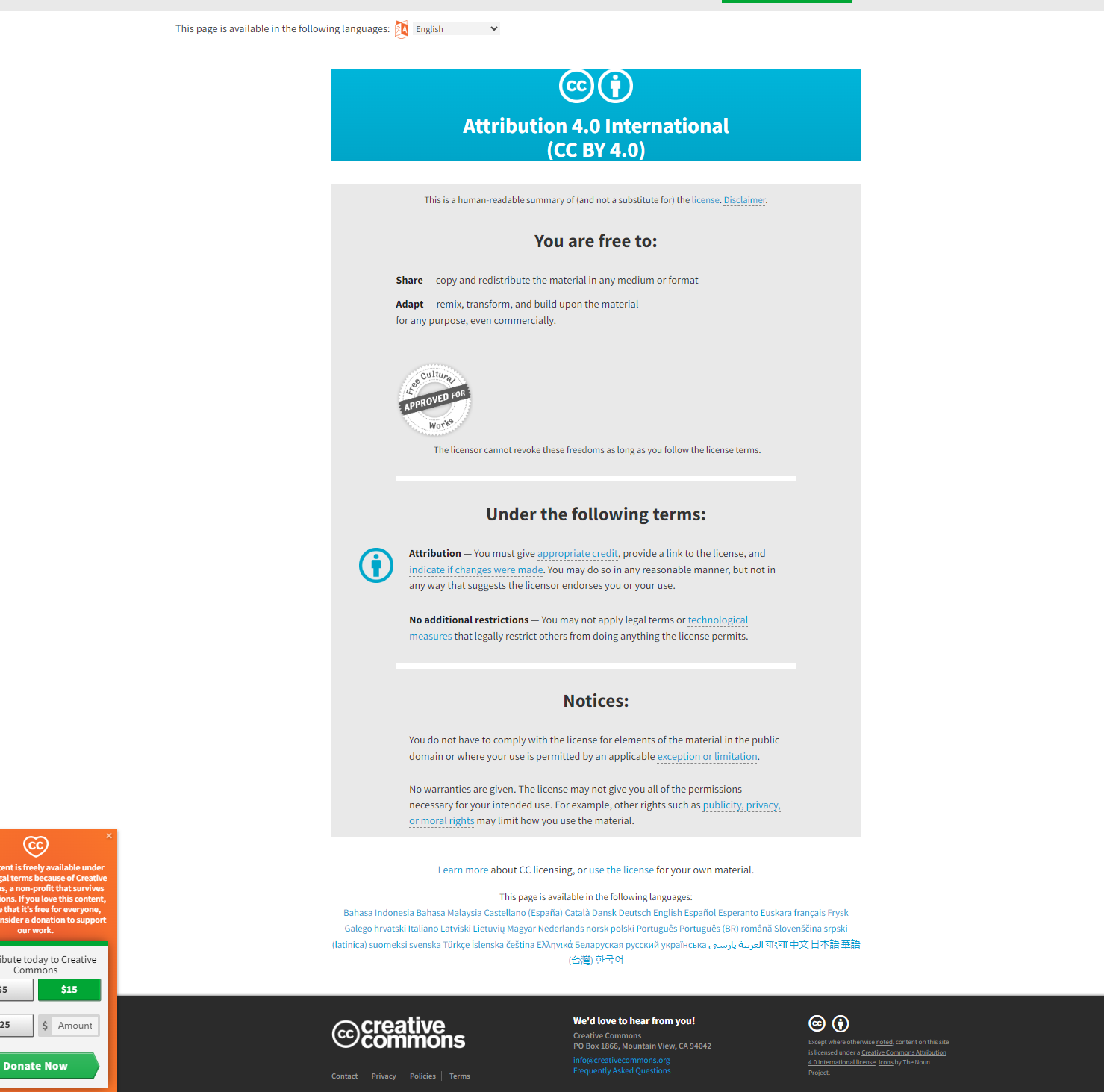

Supplement: rbae048_Supplementary_Data [file rbae048_supplementary_data.zip › Supporting informations.docx]
