# Supplementary material for: Application of magnetism in tissue regeneration: recent progress and future prospects
Source: Regen Biomater. 2024 May 7;11:rbae048. doi: 10.1093/rb/rbae048 (PMC11208728; doi:10.1093/rb/rbae048)
Supplement: rbae048_Supplementary_Data [file rbae048_supplementary_data.zip › Response to Reviewers.docx]

**Revision Summary**

**Dear Reviewers and Professor** **Nicholas Peppas:**

Thank you for your kind comments concerning our manuscript entitled **“Application of Magnetism in Tissue Regeneration: Recent progress and Future Prospects”. (Ms. No.:** **RB-2024-044)**. Those comments are all valuable and very helpful for revising and improving our paper, as well as the important guiding significance to our researches. We have studied comments carefully and made substantial correction in the revised manuscript. The main corrections in the paper and the responses to the Editor’s and Reviewer’s comments are as follows:

**(The** Blue font **is the Editor and Reviewer’s comments, and the** Red font **is our answers. Thank you)**

**Reviewer Comments to the Author**

Reviewer 1:

Comments to the Author

The authors presented a comprehensive summary of magnetism for tissue engineering, and discussed the problem and future remarks. This review would attract broad attention in the research field of tissue engineering. However, there are several issues that should be improved. Detailed comments are listed as follows:

1. The authors may present a more attractive Graphical Abstract.

Answer：Thank you very much for your comments. We've further optimized the Graphical Abstract.

2. The quality of all the figures should be improved.

Answer：Thank you very much for your comments. We've improved the image quality.

**Figure 1. Biological effects of magnetic fields in tissue engineering.**

**Figure 2. Classification of common MFs.**

**Figure 3.** **Effects of MFs on cells and tissues.**

**Figure 4. Effects of magnetic materials on cells and tissues.**

**Figure 5. MFs and magnetic materials work together to regulate cell behavior.**

**Figure 6.** **Synergistic effects of MFs and magnetic materials on tissue regeneration.**

3. In Conclusion and Perspective part, the authors may present more detailed conclusion and insightful perspective.

Answer：Thank you very much for your comments. We've made further additions and refinements to this section. Such as” Modulation of cellular functions through magnetic materials activates important pathways involved in tissue regeneration and provides directional guidance for tissue regeneration, which is an advantage over other strategies. Therefore, further research is needed to design magnetic materials that can be precisely controlled to differentiate between treating healthy and affected tissues, however current magnetic stimulation protocols struggle to effectively differentiate between injured tissues for precise stimulation. New computational models and measurement methods are expected to achieve full-time and spatial recording in the operation process, and the rise of artificial intelligence(AI) also provides new ideas for the regulation of magnetic fields. The further development and application of miniature magnetic robots is also expected to achieve molecular-level manipulation in vivo. Another major limitation related to the application of magnetic materials is the lack of biodegradability of conventional magnetic materials (iron, nickel, cobalt, etc.), which limits their potential application in regenerative medicine, and usually needs to be combined with degradable biomaterials in order to change the chemical properties of the material, but at the same time, the magnetic responsiveness of the material may be compromised. Rapid clearance of MNPs is also crucial after their role in the organism. Their prolonged stay may trigger a range of unintended reactions. In order to facilitate clinical applications, further reducing the size of MNPs or exploring new magnetic nanomaterials that are easily degradable may be potential solutions.”

4. ”In vitro experiments, the hydrogel significantly increased the length of DRG axons”, a word ‘In’ should be added at the beginning of the sentence.

Answer：Thank you very much for your comments. We have already revised that sentence.“In the vitro experiments, the hydrogel significantly increased the length of DRG axons.”

5. “the magnetic particles aggregate into strings of during the gel phase along the magnetic”, the sentence is better to be checked for reading.

Answer：Thank you very much for your comments. We have already revised that sentence.“Antman-Passig et al. mixed MNPs into a collagen hydrogel and then applied an external magnetic field on them. MNPs aggregated in clusters along the direction of the magnetic field during the gel stage, leading to the orientation of collagen fibers. Neurons cultured in this hydrogel also formed an elongated directional arrangement with no effect on normal cellular activity[230].”.

6. As mentioned in ”Morphologically, the hydrated protoelastin spongy hydrogel in the presence of MNPs showed significantly smaller pore size and less swelling”. Authors should clarify what is compared to?

Answer：Thank you very much for your comments. We have already revised that sentence.“Morphologically, the hydrated protoelastin spongy hydrogel in the presence of MNPs showed significantly smaller pore size and less swelling compared to that without MNPs addition.”

7. The point of view for the “Uto et al. prepared a PCL film with …” should be rewritten, a ‘film’ and a ‘scaffold’ are both used here, what is the difference between them? A similar problem is existing for ‘Vu et al. used a magnetic-base……’ in the text.

Answer：Thank you very much for your comments. We have already revised that sentence.“Uto et al. prepared a PCL scaffold with nano-grooves, which has good adhesion to human skin.”Film is a form of scaffold. In order to avoid confusion, we have uniformly replaced “film” with “scaffold”.

8. References for the sentence of “Downstream signaling processes during or after receptor clustering can be assessed by immunohistochemistry, electrophysiology, optical imaging, biomarker analysis, and animal behavioral assays.” Should be provided in the text.

Answer：Thank you very much for your comments. We've added references here.

① Dixon AR, Bathany C, Tsuei M, White J, Barald KF, Takayama S. Recent developments in multiplexing techniques for immunohistochemistry. Expert Review of Molecular Diagnostics 2015;15:1171-1186.

②	Wu XT, Yang XY, Song LL, Wang Y, Li YM, Liu YY, Yang XW, Wang YJ, Pei WH, Li WD. A Modified Miniscope System for Simultaneous Electrophysiology and Calcium Imaging in vivo. Frontiers in Integrative Neuroscience 2021;15.

③	Larraga-Urdaz AL, Sanchez MLF, Encinar JR, Costa-Fernandez JM. Signal amplification strategies for clinical biomarker quantification using elemental mass spectrometry. Analytical and Bioanalytical Chemistry 2022;414:53-62.

④	Laing C, Blanchard N, McConkey GA. Noradrenergic Signaling and Neuroinflammation Crosstalk Regulate -Induced Behavioral Changes. Trends in Immunology 2020;41:1072-1082.

9. The design and resolution of several figures should be improved for better reading, e.g. images in Fig.2 and Fig.5, etc.

Answer：Thank you very much for your comments. We've rearranged the images.

**Figure 2. Classification of common MFs.**

**Figure 5. MFs and magnetic materials work together to regulate cell behavior.**

10. If possible, subtitles are suggested to be supplied in section 4 and section 5.

Answer：Thank you very much for your comments. We've supplied subtitles.

4.1 Effects of SMFs on cells and tissues

4.2 Effects of PMFs on cells and tissues

4.3 Effects of RMFs on cells and tissues

5.1 Effects of MNPs on cells and tissues

5.2 Effects of magnetic composites on cells and tissues

5.3 MNPs for cell labeling

11. The format of all references should be uniformed according to the journal request.

Answer：Thank you very much for your comments. We have changed the reference format as required by the official website.

**Reviewer: 2**

Comments to the Author

This review summarizes the definition, classification, and latest progress of magnetic fields (MFs) and magnetic materials in tissue engineering. It also explores the differences and potential applications of MFs in different tissue cells, aiming to connect the applications of magnetism in various subfields of tissue engineering and provide new insights into the use of magnetism in tissue regeneration. The manuscript is well prepared, and the recent progress of magnetism in tissue engineering is systematically summarized, providing a meaningful reference for the related researchers. Thus, the topic is exciting for tissue engineering and suitable for publication after addressing the following minor issues.

1. ‘However, this approach presents challenges, such as issues related to donor sourcing, ethical concerns, and the risk of immune rejection, which need urgent attention’, sentence should be reorganized.

Answer：Thank you very much for your comments. We have reorganized the sentence. “However, this approach raises some issues that require urgent attention, such as the source of donations, ethics, immune exclusion, etc.”

2. ‘The third strategy involves repairing damaged tissues and organs through tissue engineering materials or drugs’, what is the meaning of drugs here? The authors should clarify it here.

Answer：Thank you very much for your comments. The drugs here refer to bioactive molecules that have a positive effect on tissue regeneration, such as anti-inflammatory, antibacterial, and promoting cell growth. We have reorganized the sentence. “The third strategy involves repairing damaged tissues and organs through tissue engineering materials or drugs (bioactive molecules that have a positive effect on tissue regeneration, such as anti-inflammatory, antibacterial, and promoting cell growth).”

3. Line 36, Page 4, ‘interest’ should be replaced by ‘attention’.

Answer：Thank you very much for your comments. We have reorganized the sentence. ” Given that biological substances exhibit low conductivity and negligible magnetic permeability, the application of MFs to transmit signals to cells deep within the body has gained attention”.

4. ‘Reference for the point of view ‘MFs strength and direction are typically determined by a Gaussian meter, and measurements and visualization of three-dimensional radial and vector MFs distributions based on magnetic computed tomography (CT) methods have been reported.’ should be provided.

Answer：Thank you very much for your comments. We've added references here.

① Nishimura T, Miyamoto Y, Yamada S, Iwahara M. Measurement and visualization of three-dimensional radial and vectored magnetic field distribution by use of the magnetic CT method. Ieee Transactions on Magnetics 2005;41:3739-3741.

5. The full spelling of all abbreviations in the text should be given when appearing for the first time, e.g., FDA, AC, ALP, etc. The whole manuscript should be checked for the same problem.

Answer：Thank you very much for your comments. We've checked and revised the abbreviations in the text.

6. ‘However, these stimulation methods are non-cell type specific and have limited spatial resolution’, could you provide detailed information on the spatial resolution difference?

Answer：Thank you very much for your comments. It is very difficult for a magnetic field to be confined to a very small range. At present, the spatial resolution of magnetic stimulation is only centimeter-level. But mechanical stimulation can reach cellular manipulation at the molecular level. We've added its specific resolution in the sentence. “However, these stimulation methods are non-cell type specific and have limited spatial resolution (centimeter level)”.

7. ‘The size ratio of Fig. 1B is not proper compared with others, and please enlarge the size of Fig. 1B to make it more straightforward for reading.

Answer：Thank you very much for your comments. We've changed the image size.

**Figure 1. Biological effects of magnetic fields in tissue engineering.**

8. ‘The VMFs and HMFs generated by permanent magnets are more usually used’, ‘commonly’ can be used instead of ‘usually’ here.

Answer：Thank you very much for your comments. We have reorganized the sentence. “In tissue engineering, the VMFs and HMFs generated by permanent magnets are more commonly used”.

9. ‘During the experiment, the required field strength magnitude and direction can be easily obtained by adjusting the relative position with the field source.’ The relative position of what?

Answer：Thank you very much for your comments. We have reorganized the sentence. “During the experiment, the required field strength magnitude and direction can be easily obtained by adjusting the relative position of the target to the field source”.

10. ‘AMF can exert oscillation force on magnetic materials, and can heat ferromagnetic materials and alloys such as iron, cobalt, nickel, paramagnetic materials and alloys such as aluminum, lithium, magnesium, graphite’ should be checked and rewritten.

Answer：Thank you very much for your comments. We have reorganized the sentence. “Both PMF and AMF can exert oscillating forces on magnetic materials and can heat materials with high electrical conductivity, such as iron, copper, and aluminum”.

11. What is the meaning of (B) in the sentence ‘The magnetomechanical torque is proportional to the saturation magnetization strength (Ms) and the applied MF strength (B)’？

Answer：Thank you very much for your comments. "B" is the unit of magnetic field strength. For consistency, we've replaced it with "H". “The magnetomechanical torque is proportional to the saturation magnetization strength (Ms) and the applied MF strength (H) ”.

12. ‘magnetic particles must have a high MS’. MS should be Ms here.

Answer：Thank you very much for your comments. We've changed the word.” magnetic particles must have a high Ms”.

13. ‘these methods have fast reaction speed and simple operation, but the load is not stable enough’, what is the load here? The sentence should be further clarified.

Answer：Thank you very much for your comments. We have reorganized the sentence. “these methods have fast reaction speed and simple operation, but the binding to the MNPs is not strong enough”.

14. ‘other aspect’ in the text should be avoided and deleted.

Answer：Thank you very much for your comments. We have deleted ‘other aspect’.

15. ‘and some modification methods may have a certain impact on the magnetic properties of MNPs’, what is the impact in detail? Positive or negative?

Answer：Thank you very much for your comments. We have reorganized the sentence. ”and some modification methods may reduce the magnetic responsiveness of MNPs”.

16. Fig. 3 is suggested to be rearranged. The panels in this figure are not clear for reading.

Answer：Thank you very much for your comments. We have reorganized the figure.

**Figure 3.** **Effects of MFs on cells and tissues.**

17. ‘, 0.02 T might affected’, affected should be affected.

Answer：Thank you very much for your comments. We have reorganized the sentence.“while a variable MF of 6 Hz, 0.02 T might affect central nerve regeneration”.

18. ‘studies have found that the 0.4 T rotating non-uniform RMF exposure’ should be ‘studies have found that the rotating non-uniform RMF exposure with 0.4 T’

Answer：Thank you very much for your comments. We have reorganized the sentence.” studies have found that the rotating non-uniform RMF exposure with 0.4 T”

19. Reorganize the sentence ‘Molecules such as ROS in cells and the directivity of organisms such as pigeons are obviously regulated by MFs. But these regulations have species, tissue, and even cell-specific.’

Answer：Thank you very much for your comments. We have reorganized the sentence.” Organisms such as pigeons and molecules such as ROS are regulated by MFs, but this regulation is specific”.

20. ‘There are also reports that say these…’, sentence should be rewritten.

Answer：Thank you very much for your comments. We have reorganized the sentence. “It has also been reported that MNPs can promote axonal outgrowth by activating mitogen-activated protein kinase signaling pathways”

21. ‘and the mechanisms involved are shown in Fig. 4D’, what is the mechanism here?

Answer：Thank you very much for your comments. We have added a detailed explanation. “Cells uptake MNPs into lysosomes through endocytosis, from which iron is subsequently released, and the Fenton reaction and Haber-Weiss reaction occur. These reactions lead to the formation of hydroxyl radicals, which induce cell damage.”

22. ‘hour’ and ‘h’ should be uniformed.

Answer：Thank you very much for your comments. We have uniformly changed to "h".

23. The recently published review or research articles should be discussed in the revision, for example, 10.34133/bmef.0007, 10.34133/bmef.0004, 10.1002/bmm2.12021, 10.1002/EXP.20210170, 10.1002/EXP.20210043, 10.1002/mba2.49, 10.1002/adma.202302997.

Answer：Thank you very much for your comments. These articles have been referenced and discussed in this article.

24. A native professional English speaker should polish the manuscript.

Answer：Thank you very much for your comments. We have checked and corrected the grammar in the manuscript by asking for help from an English speaker.

**Reviewer: 3**

Comments to the Author

Tissue regeneration is a hot topic in the field of biomedical research in this century. This review summarizes the definition, classification, and latest progress of MFs and magnetic materials in tissue engineering. It also explores the differences and potential applications of MFs in different tissue cells, aiming to connect

the applications of magnetism in various subfields of tissue engineering and provide new insights for the use of magnetism in tissue regeneration. I think it can be published after addressing my concerns in this regard.

1. Please add limitations to use of the magnetic materials in tissue engineering.

Answer：Thank you very much for your comments. We have further added the limitations of magnetic materials to the conclusion section. “Therefore, further research is needed to design magnetic materials that can be precisely controlled to differentiate between treating healthy and affected tissues, however current magnetic stimulation protocols struggle to effectively differentiate between injured tissues for precise stimulation. Another major limitation related to the application of magnetic materials is the lack of biodegradability of conventional magnetic materials (iron, nickel, cobalt, etc.), which limits their potential application in regenerative medicine, and usually needs to be combined with degradable biomaterials in order to change the chemical properties of the material, but at the same time, the magnetic responsiveness of the material may be compromised.”

2. Please add sentences regarding use of magnetic materials in clinical trials to the perspective section.

Answer：Thank you very much for your comments. We have further added the use of magnetic materials in clinical trials to the conclusion section. “In clinical therapy, magnetic materials are widely used in the fields of bioimaging, magneto-thermal therapy, and magnetic target guidance, but the design requirements of magnetic materials vary greatly from one treatment strategy to another. In the magnetothermal therapy system used for tumor tissues, it is necessary to focus on the limitations of magnetic materials such as the magneto-thermal conversion efficiency and the depth of tissue penetration. When targeted drug delivery is required, magnetic materials need to have a large specific surface area and ligand coupling properties, and can also work synergistically with other smart-responsive materials, such as thermosensitive polymers that can be magneto-thermally affected for drug release. “

3. Please add a comprehensive table regarding use of magnetic materials in tissue engineering.

Answer：Thank you very much for your comments. We have added the relevant table to the manuscript.

**Table 1.** **Application of magnetic materials in tissue engineering.**

| **Materials** | **Tissue** | **Effect** | **Ref** |
| --- | --- | --- | --- |
| **magnetic silk fibroin protein** | **bone** | **no toxic to osteogenic cells, improve cell adhesion and proliferation** | **[76]** |
| **polymethyl methacrylate、drug-loaded magnetic phase change nanoparticles** | **bone** | **magnetic targeting, magnetic hyperthermia through minimally invasive injection** | **[77]** |
| **superparamagnetic iron oxide nanoparticles (SPIONs)、bioactive glass** | **bone** | **regenerate the damaged bone, magnetic hyperthermia** | **[78]** |
| **MgFe_2_O_4_** | **bone** | **improve MC3T3-E1 cells adhesion and proliferation, promote ALP activation and calcium deposition, improve osteogenic ability** | **[79]** |
| **SPIONs loaded into chitosan, collagen, hyaluronic acid and calcium phosphates** | **bone** | **improve cell viability, promote tissue regeneration, have similar/equal cell(SaOS-2) behaviour activity compared with the TCP control.** | **[80]** |
| **Fe_3_O_4_ nanoparticles、magnesium calcium phosphate bone cement** | **bone、vascular** | **promote the osteogenic differentiation and mineralization of BMSCs, facilitate angiogenesis** | **[81]** |
| **magnetic cellular spheroids** | **vascular** | **assemble the cells and fuse into the vascular tissue structure** | **[82]** |
| **magnetic bacterial cellulose** | **vascular** | **facilitate angiogenesis, prevent inflammation and thrombosis** | **[83]** |
| **RGD peptide-grafted magnetic bacterial cellulose** | **vascular** | **promotes cell adhesion and proliferation, regulate endothelialization** | **[84]** |
| **magnetite cationic liposomes** | **vascular** | **prepare angiogenesis cell sheets, facilitate angiogenesis** | **[85]** |
| **magnetic silk fibroin protein** | **vascular** | **excellent biocompatibility and the growth promotion effect on CD34-labeled vascular endothelial cells** | **[86]** |
| **magnetic responsive hydrogel of methacrylated chondroitin sulfate** | **tendon、bone** | **affect cell morphology as well as expression and synthesis of tendons and bone-like matrix** | **[87]** |
| **magnetically-responsive nanocomposite hydrogel** | **tendon** | **promote cell elongation and alignment** | **[88]** |
| **hybrids of cellulose nanocrystals decorated with MNPs** | **tendon** | **promote cell alignment, promote tendon sheath production of human adipose stem cells** | **[89]** |
| **MNPs** | **tendon** | **stimulate the overall expression of tendon-associated genes and the deposition of tendon-associated proteins** | **[90]** |
| **MNPs、starch and polycaprolactone** | **tendon** | **promote tendon componentization of ASCs under magnetic stimulation conditions** | **[91]** |
| **magnetic spongy-like hydroge** | **tendon** | **support human tendon-derived cell growth, adhesion, diffusion, and migration** | **[92]** |
| **biohybrid microswimmers** | **muscle** | **enable precise movement to a single C2C12-derived myotube, trigger myotube contraction** | **[93]** |
| **remote magnetic short nanofibers** | **muscle** | **increase the arrangement of muscle fibers, enhance skeletal muscle regeneration** | **[94]** |
| **alginate-coated magnetic microparticles** | **muscle** | **promote myoblast alignment** | **[95]** |
| **magnetic bioreactor system、magnetic hydrogel** | **muscle** | **accelerate the differentiation of mouse myoblasts in hydrogels, increase the diameter and length of myotubes in vitro** | **[96]** |
| **cobalt based titanium dioxide (Co-doped TiO_2_) nanofibers** | **muscle** | **support cell adhesion and growth of myoblasts** | **[97]** |
| **magnetic nanochains** | **nerve** | **promote the alignment of neural stem cells, promote the directed growth of neurons** | **[98]** |
| **mesoporous hollow Fe_3_O_4_ nanoparticles** | **nerve** | **induce polarization of macrophages, promote the proliferation of neural stem cells and the migration of vascular endothelial cells** | **[99]** |
| **SPIONs** | **nerve** | **promote nerve regeneration by inducing and maintaining the repaired phenotype of Schwann cells** | **[100]** |
| **self-rectifying magnetoelectric metamaterial** | **nerve** | **convert magnetic stimulation to electrical stimulation and wireless stimulation of peripheral nerves, restore sensory reflexes** | **[101]** |
| **magnetic poly(lactic-co-glycolic acid) (PLGA) microcapsules** | **nerve** | **regulate the release of NGF, promote neurite extension** | **[102]** |

4. GA must be replaced with an eye catching scheme.

Answer：Thank you very much for your comments. We have redrawn GA.

**Reviewer: 4**

Comments to the Author

This review provides a comprehensive report on the definition, classification, and latest progress of magnetic fields (MFs) and magnetic materials in tissue engineering. It provides new insights for magnetism and magnetic materials in tissue regeneration through exploring the interaction with tissues and cells. It is interesting and well-written. The authors are requested to address the following minor concerns to further improve the manuscript.

1. Tissue engineering scaffold is one of the important factors in tissue regeneration, and attention should also be paid to the effect of magnetic fields on magnetic materials and their corresponding changes, such as degradation of biomaterials, micro/nano deformation of biomaterials caused by magnetic fields, which further affects the morphology or function of cells. So literature in this area is worth paying attention to, such as Nanoscale, 2023, 15, 4123; J Mater Chem B, 2022, 10(38): 7808-7826; Advanced Functional Materials, 2021, 31, 2009661; Nanoscale, 2019, 11, 23423-37, etc.

Answer：Thank you very much for your comments. These articles have been referenced and discussed in this article.

2. The authors are requested to provide a detailed discussion on the possible problems, promises, and solutions for the future development of this field.

Answer：Thank you very much for your comments. We have added further discussion in the corresponding discussion section and conclusion section of the manuscript, such as “When a magnetic field is applied, it often brings complex stimuli such as mechanical stimulation, thermal stimulation, and magnetic stimulation. The mechanism by which various stimuli interact with each other is difficult to fully elucidate. In addition, the surface properties of the material also have a non-negligible effect on the surrounding cells. There is still a huge gap to be filled in this field, which may also be a future research direction.”

“Modulation of cellular functions through magnetic materials activates important pathways involved in tissue regeneration and provides directional guidance for tissue regeneration, which is an advantage over other strategies. Therefore, further research is needed to design magnetic materials that can be precisely controlled to differentiate between treating healthy and affected tissues, however current magnetic stimulation protocols struggle to effectively differentiate between injured tissues for precise stimulation. New computational models and measurement methods are expected to achieve full-time and spatial recording in the operation process, and the rise of artificial intelligence(AI) also provides new ideas for the regulation of magnetic fields. The further development and application of miniature magnetic robots is also expected to achieve molecular-level manipulation in vivo. Another major limitation related to the application of magnetic materials is the lack of biodegradability of conventional magnetic materials (iron, nickel, cobalt, etc.), which limits their potential application in regenerative medicine, and usually needs to be combined with degradable biomaterials in order to change the chemical properties of the material, but at the same time, the magnetic responsiveness of the material may be compromised. Rapid clearance of MNPs is also crucial after their role in the organism. Their prolonged stay may trigger a range of unintended reactions. In order to facilitate clinical applications, further reducing the size of MNPs or exploring new magnetic nanomaterials that are easily degradable may be potential solutions.”

3. Some upcoming techniques/applications related to magnetic fields or magnetic materials can be added to the future prospective section. This will make the section more extensive.

Answer：Thank you very much for your comments. We have further added new technologies to the manuscript that may be coming.” New computational models and measurement methods are expected to achieve full-time and spatial recording in the operation process, and the rise of AI also provides new ideas for the regulation of magnetic fields. The further development and application of miniature magnetic robots is also expected to achieve molecular-level manipulation in vivo”.” In order to facilitate clinical applications, further reducing the size of MNPs or exploring new magnetic nanomaterials that are easily degradable may be potential solutions”.” In addition, magnetic manipulation of cells or molecules is currently mostly limited to a single magnetic material. By changing the composition or particle size to obtain MNPs with different magnetic responsiveness, and labeling different cells or peptides in a non-uniform magnetic field, it is expected that more precise assembly of cells and even biological macromolecules can be realized with the further improvement of magnetic field accuracy”.

**Review Editor**

Comments to the Author:

This manuscript is of initial interest to the Board but will require significant revisions before the potential publication. Please submit a revised version which will be reviewed again.

Meanwhile, the authors should revise the graphic abstract (GA). Last time, the authors have enlarged the font size, but there is too much empty space especially on the margin of the new GA. Please do not leave blank space on the edge of GA.

Here are some examples for you:

https://academic.oup.com/rb/article/doi/10.1093/rb/rbac098/6873888

https://academic.oup.com/rb/article/doi/10.1093/rb/rbac007/6522134

https://academic.oup.com/rb/article/doi/10.1093/rb/rbac078/6760268

https://academic.oup.com/rb/article/doi/10.1093/rb/rbac049/6648358

https://academic.oup.com/rb/article/doi/10.1093/rb/rbad035/7114568

https://academic.oup.com/rb/article/doi/10.1093/rb/rbad056/7189747

https://academic.oup.com/rb/article/doi/10.1093/rb/rbad112/7470753

GA will be eventually shown along with the abstract in the PDF file for all of publications in the journal Regenerative Biomaterials since 2022. Please further click the button of "PDF". You can see that GA is important as a part of the paper. It should be designed and shown exactly with the due size (3.6 cm ×8.4 cm) and is expected to be impressive. The preferred font type of text in the GA is Arial, no smaller than 8 pt. You can NOT show GA in any magnified way in order to guarantee that every element in GA of the due size is distinguishable. You should not purposely leave empty space on either side of GA to reach the required size.

GA could be the key 'results’ image from the paper itself, or a specially designed figure that captures the key takeaway message of the paper and enables the viewer to understand what the paper is about. The latter is more encouraged. It does not have to replicate all the information in the text abstract, or be a comprehensive account of the paper. Although allowed, it is not encouraged to simply copy a whole graphic already used in the text of the manuscript.

Answer：Thank you very much for your comments. We have redrawn GA.

Once again, we appreciate the Editor and Reviewer’s consideration and comments on our manuscript very much.

We have tried our best to improve the manuscript according to the Reviewer’s comments. Besides, we also made some other little changes in red in the manuscript without affecting the content and framework of the paper.

We appreciate for Editors/Reviewers’ warm work earnestly, and hope that the

correction will meet with approval.

Once again, thank you very much for your comments and suggestions.

Guicai Li
